# Drug-induced lactate confers ferroptosis resistance via p38-SGK1-NEDD4L-dependent upregulation of GPX4 in NSCLC cells

**DOI:** 10.1038/s41420-023-01463-5

**Published:** 2023-05-15

**Authors:** Feng Cheng, Jintao Dou, Yi Yang, Shaojie Sun, Ruiqi Chen, Zhijian Zhang, Huijun Wei, Jianhui Li, Zhihao Wu

**Affiliations:** 1 Research laboratory of Tumor Microenvironment, Wannan Medical College, 241001 Wuhu, China; 2 School of Anesthesiology, Wannan Medical College, 241001 Wuhu, China; 3 School of Pharmacy, Wannan Medical College, 241001 Wuhu, China; 4 School of Medical Imageology, Wannan Medical College, 241001 Wuhu, China; 5 School of Stomatology, Wannan Medical College, 241001 Wuhu, China; 6 Anhui Province Key laboratory of Active Biological Macro-molecules Research, Wannan Medical College, 241001 Wuhu, China; 7 Provincial Engineering Laboratory for Screening and Re-evaluation of Active Compounds of Herbal Medicines in Southern Anhui, Wannan Medical College, 241001 Wuhu, China; 8 Key Laboratory of Non-coding RNA Transformation Research of Anhui Higher Education Institution, Wannan Medical College, 241001 Wuhu, China

**Keywords:** Non-small-cell lung cancer, Ubiquitylation, Cancer metabolism, Cancer therapeutic resistance

## Abstract

Ferroptosis is a newly defined non-apoptotic programmed cell death resulting from the accumulation of lipid peroxides. Whether ferroptosis plays any role in chemotherapy remains to be established. Here, we reported that ferroptosis represents a part of the chemotherapeutic drug etoposide-induced cell death response in Small Cell Lung Cancer (SCLC) cells and adaptive signaling molecule lactate protects Non-Small Cell Lung Cancer (NSCLC) from etoposide-induced ferroptosis. Lactate derived from metabolic reprogramming increases the expression of glutathione peroxidase 4 (GPX4) to promote ferroptosis resistance in NSCLC. Furthermore, we identified E3-ubiquitin ligase NEDD4L as a major regulator of GPX4 stability. Mechanistically, Lactate increases mitochondrial ROS generation and drives activation of the p38-SGK1 pathway, which attenuates the interaction of NEDD4L with GPX4 and subsequent ubiquitination and degradation of GPX4. Our data implicated the role of ferroptosis in chemotherapeutic resistance and identified a novel post-translational regulatory mechanism for the key Ferroptosis mediator GPX4.

## Introduction

The ability of cancer cells to resist chemotherapy-induced cell death is a problem of paramount importance in cancer therapy. It was once thought that almost all cell death induced by cancer therapy resulted from the activation of caspase-dependent apoptosis. More recent studies have implicated a novel mode of regulated cell death, termed ferroptosis, in response to radiotherapy and immunotherapy [1, 2]. Ferroptosis is a new form of regulated cell death characterized by the production of iron-dependent reactive oxygen species (ROS) that causes plasma membrane peroxidation [3, 4]. An initial examination revealed that ferroptosis was morphologically and genetically distinct from apoptosis and cannot be rescued by biochemical inhibitors of apoptosis such as Z-vad-fmk [5]. Recently, the detailed characterization of ferroptosis regulators has identified glutathione peroxidase 4 (GPX4) as the key enzyme that protects cells from ferroptosis [6]. Unlike other GPXs, GPX4 is the only antioxidant enzyme that acts on phospholipid to prevent detrimental lipid hydroperoxides. Hence, pharmacological inhibitors of GPX4, such as RSL3, are used as specific inducers of ferroptosis. Furthermore, since GPX4 uses glutathione (GSH) as a co-factor to reduce lipid hydroperoxides, depletion of GSH also contributes to ferroptotic cell death resulting from the loss of GPX4 activity [7, 8]. In addition to inhibiting GPX activity, regulation of GPX4 expression or stability remains poorly understood.

NEDD4L (also known as NEDD4-2, neural precursor cell expressed developmental down-regulated protein) is a HECT E3 ubiquitin ligase composed of the phospholipid binding (C2) domain, 4 WW domains that interact with the PY motifs (L/PPXY) of target proteins, and a ubiquitin ligase HECT domain at the carboxyl terminus [9]. The best characterized NEDD4L targets are membrane proteins including ENaC (epithelial sodium channel) and CFTR (Cystic fibrosis transmembrane conductance regulator) [10, 11]. NEDD4L WW domains bind these membrane proteins PY motifs targeting them for degradation. Recent reports indicated that the binding of NEDD4L to its substrate proteins was controlled by serum-glucocorticoid-regulated kinase 1 (SGK1) [11]. SGK1-mediated phosphorylation of NEDD4L on serine 448 prevent the interaction of NEDD4L with its target proteins, such as ENaC. The NEDD4L has now been shown to play a crucial role in maintaining membrane protein homeostasis, wherein ferroptosis was attributed to lipid peroxidation induced by a dysfunctional membrane-specific antioxidant GPX4, raising the possibility that a function link may exist between NEDD4L and GPX4. However, no direct experimental evidence for this link has yet been examined.

Lung cancer remains the leading cause of cancer deaths worldwide with non-small cell lung cancer (NSCLC) accounting for 80% cases. Despite the growing arsenal of therapies, such as targeted therapy and immunotherapy, systemic chemotherapy is still the mainstay for lung cancer [12]. However, various histological types of lung cancer demonstrate different sensitivities to chemotherapeutic drugs. Etoposide, a classical chemotherapy drug commonly used to treat lung cancer, has been shown to have more efficacy and sensitivity towards small cell lung cancer (SCLC) than NSCLC [13, 14]. Unfortunately, the underlying molecular mechanisms remain unclear. A better understanding of chemotherapy sensitivity could greatly improve lung cancer treatment.

Even in the aerobic state, tumor cells still carry out their primary energy supply through glycolysis, known as the Warburg effect [15, 16]. Notably, high levels of lactate, a metabolite of glycolysis, accumulate in the tumor microenvironment, with concentrations as high as 40 mM. Although historically viewed as metabolic waste in the past, numerous studies have since revealed that lactate is vital for tumor metastasis, immune escape and drug resistance [17–20]. However, its role in ferroptosis in cancer cells remains largely unexplored.

Here, we report for the first time that the difference in etoposide sensitivity between different type of lung cancer cells is partly due to its resistance to ferroptosis, which is mediated by GPX4, a key enzyme controlling lipid peroxide homeostasis. Further evidence suggests that lactic acid generated by metabolic reprogramming promotes the accumulation of GPX4 and leads to etoposide resistance in NSCLC cells. Mechanistically, lactate is transported from extracellular to intracellular compartments, activating SGK1-NEDD4L through mitochondrial ROS to upregulate GPX4 expression. Our findings provide insights into the molecular basis of ferroptosis and highlight potential avenues for improving the sensitivity of chemotherapy in NSCLC.

## Results

### GPX4 dictates sensitivity to ferroptosis induction by etoposide in lung cancer cells

**Fig. 1:**
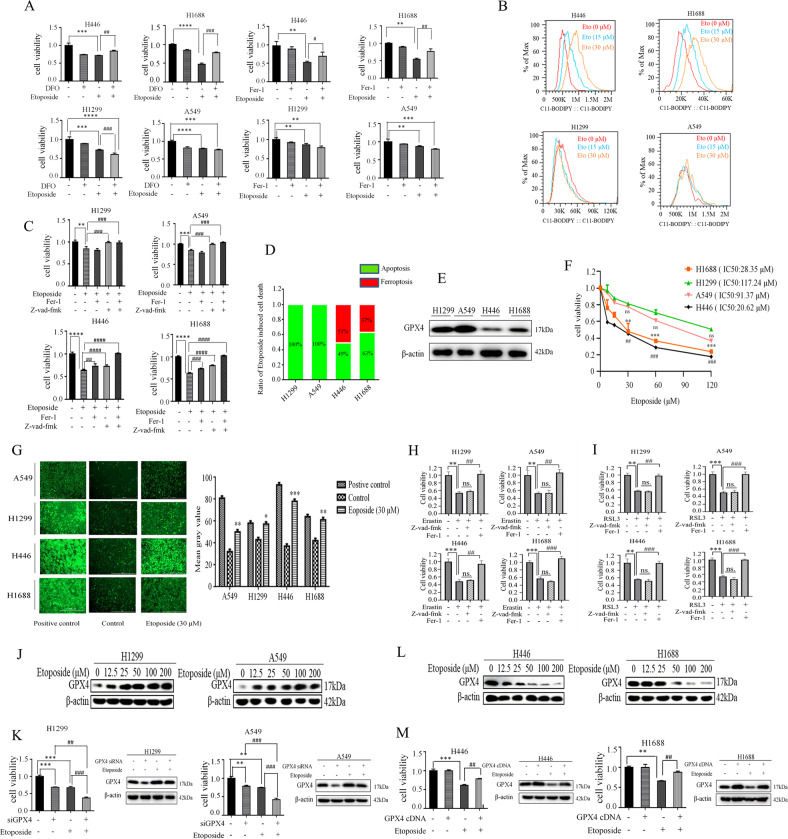
GPX4 dictates sensitivity to ferroptosis induction by etoposide in lung cancer cells. **A** H446, H1688, A549, and H1299 were untreated, or treated with DFO (100 µM), Fer-1 (1 µM), Etoposide (25 µM) and DFO (or Fer-1) plus Etoposide. MTT assay was used to detect cell viability after 24 h of continuous culture. The bars represent the mean ± S.D. of triplicates. (*p < 0.05, **p < 0.01, ***p < 0.001; for difference from the control group. ^#^*p* <0.05, ^##^*p* < 0.01, ^###^*p* < 0.001 for difference from the treatment group by ANOVA with Dunnett’s correction for multiple comparisons). **B** Indicated cells were treated with Etoposide (0 µM, 15 µM, and 30 µM) for 24 h, and the lipid ROS level was assessed by flow cytometry using C11-BODIPY. **C** Cells were untreated, or treated with Etoposide (25 µM), Etoposide plus Fer-1(1 µM), Etoposide plus Z-vad-fmk (40 µM) or Etoposide plus Fer-1 plus Z-vad-fmk. The cell viability was determined by MTT at 24 h time point (**p < 0.01, ***p < 0.001; for difference from the control group. ^###^*p* < 0.001, ^####^*p* < 0.0001, for difference from the treatment group by ANOVA with Dunnett’s correction for multiple comparisons.) . **D** Detailed cell death induced by etoposide in lung cancers. Ferroptosis is defined as the percentage of cell death rescued by Fer-1, Apoptosis is defined as the percentage of cell death rescued by z-vad-fmk in Etoposide-induced cell death. **E** The basal levels of GPX4 in different lung cancer cell lines. **F** cells were treated with different concentrations of Etoposide, the cell viability was determined by MTT (*p < 0.05, **p < 0.01, ***p < 0.001, ^##^*p* < 0.01, ^###^*p* < 0.001, for difference from etoposide-treated H1299 cells by ANOVA with Dunnett’s correction for multiple comparisons, ns means no statistical difference). **G** Cells were untreated (control) or treated with positive control (a compound mixture for oxidative stress) and Etoposide (30 µM). ROS levels were analyzed by fluorescent probe DCFH-DA. (Original magnification ×100. Scale bar, 1000 μm) (left panel). Quantitative analysis of left panels (right panel). (*p < 0.05, **p < 0.01, ***p < 0.001 for difference from the control group by ANOVA with Dunnett’s correction for multiple comparisons). **H** Cells were untreated (control), or treated with Erastin (5 µM) ± Fer-1 (1 µM) or Z-vad-fmk (40 µM), and cell viability was determined by MTT at 24 h time point. (**p < 0.01, ***p < 0.001 for difference from the control group. ##*p* < 0.01, ###*p* < 0.001 for difference from the treatment group by ANOVA with Dunnett’s correction for multiple comparisons, ns means no statistical difference). **I** Cells were untreated (control), or treated with RSL3 (2 µM) ± Fer-1 (1 µM) or Z-vad-fmk (40 µM), and cell viability was determined by MTT at 24 h time point. (**p < 0.01, ***p < 0.001 for difference from the control group. ^##^*p* < 0.01, ^###^*p* < 0.001 for difference from the treatment group by ANOVA with Dunnett’s correction for multiple comparisons, ns means no statistical difference). **J** Western blot examines GPX4 expression in NSCLC cells treated with Etoposide. **K** Cells were untreated, or treated with siGPX4, Etoposide (25 µM), siGPX4 plus Etoposide and cell viability was determined by MTT at 24 h time point (left panel). Western blot analysis of GPX4 after transfection with GPX4 siRNA or control and then indicated etoposide treatment (right panel) (**p < 0.01, ***p < 0.001 for difference from the control group, ^##^*p* < 0.01, ^###^*p* < 0.001 for difference from the treatment group by ANOVA with Dunnett’s correction for multiple comparisons). **L** Western blot examines GPX4 expression in SCLC cells treated with Etoposide. **M** Cells were untreated, or treated with Etoposide or transfected with GPX4 cDNA, or GPX4 cDNA plus Etoposide and cell viability was determined by MTT at 24 h time point (left panel). Western blot analysis of GPX4 after transfection with GPX4 cDNA or vector control and then indicated etoposide treatment (right panel) (**p < 0.01, ***p < 0.001 for difference from the control group. ^##^*p* < 0.01, for difference from the treatment group by ANOVA with Dunnett’s correction for multiple comparisons).

Since etoposide is also associated with the induction of reactive oxygen species (ROS) and ferroptosis is driven by ROS-dependent lipid peroxidation. We reasoned that ferroptosis might be involved in etoposide-induced cell death in lung cancer cells. To directly assess whether etoposide could induce ferroptosis in lung cancer cells, we examined the impact of the ferroptosis inhibitors, ferrostatin-1 (Fer-1) and Deferoxamine (DFO), on the cell viability of etoposide-treated lung cancer cells. Despite recent advances in targeted therapies for distinct subtypes of lung cancer, no effective targeted therapies exist for KRAS/TP53-driven NSCLC. The systemic chemotherapy is still the main treatment regimen for KRAS/TP53-mediated NSCLC. Therefore, we chose the A549 cell line, which has an activating mutation in KRAS, and p53-deficient H1299 cells of NSCLC, H446, and H1688 cell lines of SCLC, which both contain p53 and PTEN mutations, to represent our in vitro model for lung cancer chemotherapy resistance study. Interestingly, we found marked differences in cell viability between NSCLC and SCLC cell lines in response to those ferroptosis inhibitors (Fig. 1A). In SCLC cell lines H446 and H1688, the etoposide-induced cell death was partially suppressed by Fer-1 and DFO. However, neither Fer-1 nor DFO can restore cell survival that had been reduced by exposure to etoposide in NSCLC cell lines A549 and H1299. Furthermore, etoposide-induced accumulation of lipid peroxidation, a hallmark of ferroptosis, as determined by lipid peroxidation-sensitive dye BODIPY-C11 in SCLC cell line H1688, but not in NSCLC A549 cells (Fig. 1B). To determine the extent of etoposide-induced ferroptosis in different histological lung cancer cells, the corresponding pharmacological inhibitors were used to rescue the cell viability reduced by etoposide (Fig. 1C, D). We found that addition of either Fer-1 or Z-vad-fmk, a specific inhibitor of apoptosis, only partially rescued cell death by etoposide in SCLC cell lines H446 and H1688 cells, however, combining Fer-1 with Z-vad-fmk completely suppressed etoposide-induced cell death in H446 and H1688 cells. To our surprise, only Z-vad-fmk, but not Fer-1, effectively restored cell survival under etoposide treatment in NSCLC A549 and H1299 cells, suggesting that ferroptosis represents a part of etoposide-induced cell death response in SCLC cells rather than NSCLC cells. This is further supported by the evidence that the basal levels of GPX4 among these cell lines correlated positively with the status of chemoresistance (Fig. 1E, F).

An important issue is why etoposide-induced ferroptosis is specific to SCLC cells since we consistently confirmed that ROS generation induced by etoposide was elevated in both SCLC and NSCLC cells (Fig. 1G). Intriguingly, ferroptosis was found to be induced in both SCLC and NSCLC cells by a bona fide ferroptosis inducer erastin via inhibition of system x_c_^-^ (Fig. 1H). system x_c_^-^ is a cystine-glutamate antiporter that is essential for the synthesis of the GSH and functioning of GPX4. Consistently, cell death can be induced by treatment of cells with the covalent GPX4 inhibitor RSL3 (Fig. 1I). When GPX4 is inactive, ferroptosis can be inhibited by radical trapping antioxidant Fer-1. We demonstrated that treatment of cells with Fer-1, but not with the pan-caspase inhibitor Z-vad-fmk, rescued the erastin- or RSL3-induced cell death (Fig. 1H, I), indicating ferroptosis can be effectively induced in both SCLC and NSCLC cells. Therefore, we hypothesize that the ability of NSCLC cells to suppress etoposide-induced ferroptosis might be due to the upregulated repair system in preventing detrimental phospholipid oxidation. Indeed, etoposide treatment significantly upregulated GPX4 levels in NSCLC H1299 and A549 cells in a dose-dependent manner (Fig. 1J). Furthermore, short interfering RNA (siRNA)-mediated GPX4 silencing significantly potentiated etoposide-induced cell death in H1299 and A549 cells (Fig. 1K). In contrast, GPX4 levels were dramatically decreased by etoposide in SCLC H446 and H1688 cells (Fig. 1L), and etoposide-reduced cell viability of H446 and H1688 cells was substantially mitigated by overexpression of GPX4 (Fig. 1M). Taken together, our data strongly suggest that GPX4 dictates sensitivity to ferroptosis induction by etoposide in lung cancer cells.

### Metabolic reprogramming contributes to GPX4 regulation

**Fig. 2:**
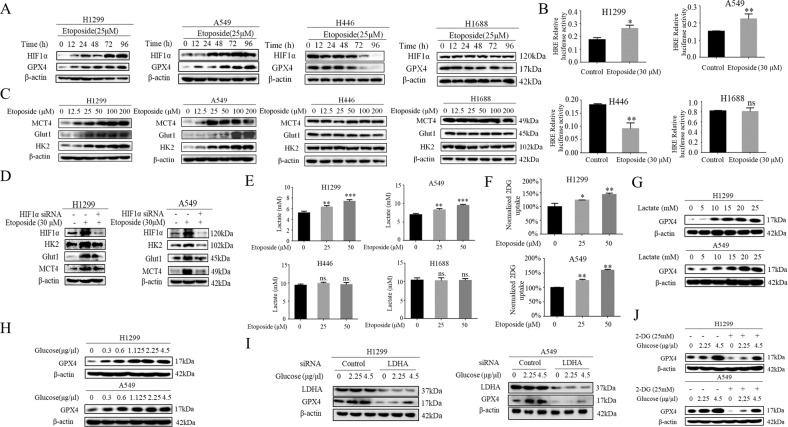
Metabolic reprogramming contributes to GPX4 regulation. **A** H446, H1688, A549, and H1299 were treated with Etoposide (25 µM) for the indicated time, Western blot was used to determine HIF-1α, GPX4 protein levels. **B** Co-transfection of the HRE promoter reporter gene with control Renilla luciferase reporter gene was done in indicated cells. 24 h later cells were treated with Etoposide (30 µM) for 24 h, and luciferase activity was measured and normalized using dual luciferase reporter system and the bars represent the mean ± S.D. of triplicates (*p < 0.05, **p < 0.01, for difference from untreated control by ANOVA with Dunnett’s correction for multiple comparisons, ns means no statistical difference). **C** Western blot examines MCT4, Glut1, HK2 expression in cells treated with different doses of Etoposide. **D** Indicated cells were untreated, or treated with Etoposide (30 µM) for 48 h and siHIF-1 α for 24 h then treated with Etoposide (30 µM) . Western blot was used to determine HIF-1 α, MCT4, Glut1, HK2 protein levels. **E** Cells were treated with Etoposide and then extracellular lactate concentration was measured by Lactate Colorimetric/Fluorometric Assay Kit (Biovision). (**p < 0.01, ***p < 0.001 for Difference from control cells by ANOVA with Dunnett’s correction for multiple comparisons, ns means no statistical difference). **F** Measurement of 2-DG uptake in cells after exposed etoposide for 5 h by Glucose Uptake Kit (*p < 0.05, **p < 0.01, for difference from untreated control by ANOVA for multiple comparison, ns means no statistical difference). **G** Western blot examines GPX4 expression in cells treated with different concentrations of lactate. **H** Western blot examines GPX4 expression in cells treated with different concentrations of glucose. **I** 48 h after transfection with control siRNA or LDHA siRNA, cells were treated with different concentrations of glucose. Western blot examines GPX4 expression. **J** Western blot analysis of GPX4 in cells treated with glucose in the presence of 2-DG.

The ability of GPX4 to protect against etoposide-induced ferroptosis in NSCLC cells has therapeutic implications for drug targeting. However, little is known about how the expression of GPX4 is regulated by chemotherapeutic agent in lung cancer cells. Our previous study has established that lactate, a metabolite of tumor metabolic reprogramming, contributes significantly to chemotherapeutic agent etoposide resistance in NSCLC rather than SCLC, hinting at a potential link of lactate to ferroptosis resistance. We next sought to determine whether lactate resulting from metabolic reprogramming regulates GPX expression. As proof of principle of the functional relevance of metabolic reprogramming, we first confirmed our previous report that etoposide strongly induced the activity of HIF-1α, a key player of metabolic reprogramming, by western blotting and luciferase reporter assay (Fig. 2A, B). Markedly, this occurred with a parallel increase in HIF-1α target genes Hexokinase 2 (HK2), glucose transport 1 (Glut1), Monocarboxylate transport 4 (MCT4) in NSCLC H1299 and A549 cells, but not in H446 and H1688 cells (Fig. 2C). The dominant role of HIF-1α in etoposide-induced metabolic reprogramming is established by the fact that siRNA-mediated HIF-1α silencing abolished the induction of HK2, Glut1 and MCT4 levels by etoposide (Fig. 2D). Importantly, the levels of extracellular lactate and glucose consumption, the hallmarks of metabolic reprogramming, are elevated only in NSCLC H1299 and A549 cells (Fig. 2E, F). We next evaluate a potential involvement of lactate in GPX4 regulation by treating H1299 and A549 cells with exogeneous lactate. Strikingly, lactate dose-dependently upregulated GPX4 levels in H1299 and A549 cells (Fig. 2G). To demonstrate physiological relevance of metabolic reprogramming in lactate-induced GPX4 expression, we showed that glucose stimulation triggered strong induction of GPX4 levels in a dose-dependent manner (Fig. 2H). Furthermore, after inhibiting metabolic reprogramming using siRNA-mediated silencing of lactate dehydrogenase (LDHA), that catalyzes the conversion of pyruvate to lactate, as well as employing a compound 2-deoxy-d-glucose (2-DG), a competitive inhibitor of hexokinase, glucose-induced GPX4 expression is largely ablated by those agents in H1299 and A549 cells (Fig. 2I, J), indicating the lactate derived from metabolic reprogramming plays a dominant role in GPX4 induction.

### E3-ubiquitin ligase NEDD4L is a major regulator of GPX4 stability

**Fig. 3:**
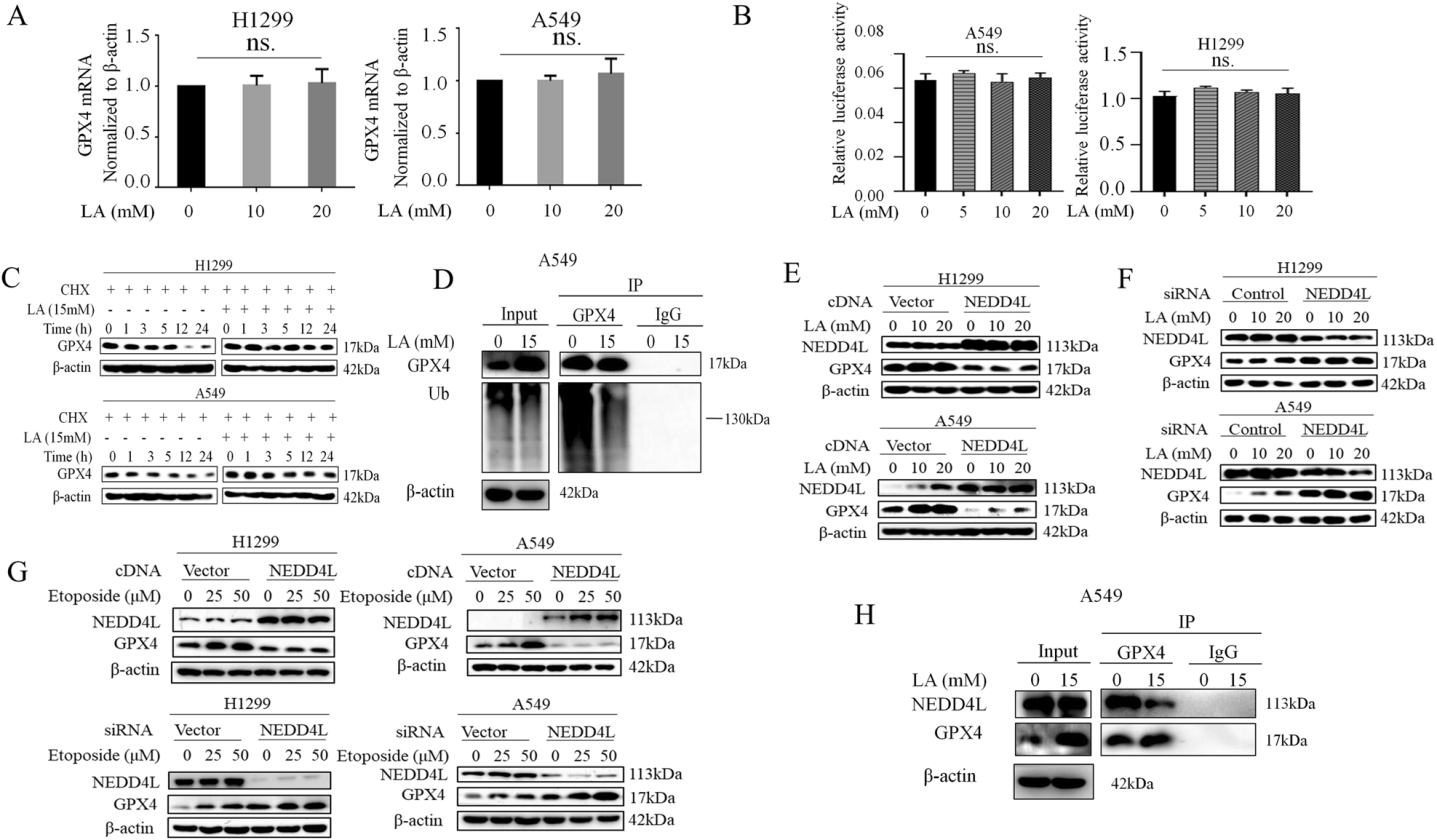
E3-ubiquitin ligase NEDD4L is a major regulator of GPX4 stability. **A** A549 and H1299 cells were treated with Etoposide (0, 25 and 50 μM) for 48 h. The levels of GPX4 mRNA were detected by real time qRT-PCR. (ns means no statistical difference by ANOVA with Dunnett’s correction for multiple comparisons). **B** A549 and H1299 cells were co-transfected with a plasmid of the GPX4 promoter luciferase reporter gene with a plasmid of control Renilla luciferase reporter gene. At 36 h after transfection, cells were treated with lactate (0, 5, 10, and 20 μM) for 3 h, and luciferase activity was detected using the dual luciferase reporter system. **C** Cells were treated with CHX (Cycloheximide, 50 μg/mL) for the indicated time in the presence or absence of 15 mM lactate. Western blot was used to determine GPX4 protein levels. **D** Cells were treated with lactate for 3 h after which cell lysates were immunoprecipitated with anti- GPX4 antibody and then Western blotted with anti-Ub. **E** After transfection with NEDD4L cDNA for 48 h, A549 and H1299 cells were treated with lactate (0, 10, and 20 μM) for 3 h. Western blot was carried out for analysis of GPX4 levels. **F** After transfection with NEDD4L siRNA for 48 h, A549 and H1299 cells were treated with lactate (0, 10 and 20 μM) for 3 h. Western blot was used for detection of GPX4 levels. **G** After transfection with NEDD4L cDNA or NEDD4L siRNA for 36 h, A549 and H1299 cells were treated with Etoposide (0, 25 and 50 μM) for 12 h. Western blot was carried out for analysis of GPX4 levels. **H** Cells were treated with lactate for 3 h after which cell lysates were immunoprecipitated with anti-GPX4 antibody and then western blotted with anti-NEDD4L.

We next wanted to understand how GPX4 expression is regulated by lactate. We first quantified the GPX4 mRNA expression in lactate-treated H1299 and A549 cells and found GPX4 mRNA levels were not changed by lactate (Fig. 3A). Similarly, the promoter activity of GPX4 as determined by luciferase reporter assay was also not affected by lactate (Fig. 3B). Thus, the results raised the possibility that lactate might promote GPX4 protein stabilization. The impact of lactate on GPX4 protein stability was examined using the protein synthesis inhibitor cycloheximide (CHX) chase assay. Indeed, GPX4 protein half-life is significantly increased in lactate-treated H1299 and A549 cells (Fig. 3C), suggesting a post-translational mechanism of regulation of the GPX4 by lactate.

Ubiquitination is a well-characterized protein modification system that regulates a wide range of protein degradation and stabilization. To determine whether the lactate-induced stabilization of GPX4 was mediated through the ubiquitination pathway, immunoprecipitation assay (IP) to detect ubiquitination levels was performed in A549 cells treated with lactate. IP result showed that GPX4 ubiquitination was significantly reduced by the addition of lactate (Fig. 3D). Insights into the mechanism of GPX4 regulation came from a proteomics study using the ScanProsite approach (www.expasy.ch/tools/scanprosite). We found that the GPX4 protein contains an LPXY sequence, similar to the membrane-located E3-ubiquitin ligase NEDD4L conserved binding PY (PPXY) motifs. To determine whether NEDD4L was involved in GPX4 ubiquitination, the cells were transfected with NEDD4L cDNA. Indeed, GPX4 levels in both H1299 and A549 cells were significantly suppressed by overexpression of NEDD4L in the presence and absence of lactate (Fig. 3E). In contrast, knockdown of endogenous NEDD4L levels by siRNA increased GPX4 protein levels markedly (Fig. 3F). Alternatively, similar results were obtained when cells transfected with either NEDD4L cDNA or siRNA were treated with etoposide (Fig. 3G). Furthermore, co-immunoprecipitation was performed to validate the interaction between GPX4 and NEDD4L. As shown in Fig. 3H, NEDD4L was readily detected in the immunoprecipitated complexes of GPX4. Strikingly, lactate treatment decreased the affinity of NEDD4L for GPX4. Taken together, these data suggested the involvement of NEDD4L in GPX4 ubiquitination and lactate treatment attenuated the interaction of NEDD4L with GPX4.

### Activation of SGK1 by lactate reduced the interaction of NEDD4L with GPX4

**Fig. 4:**
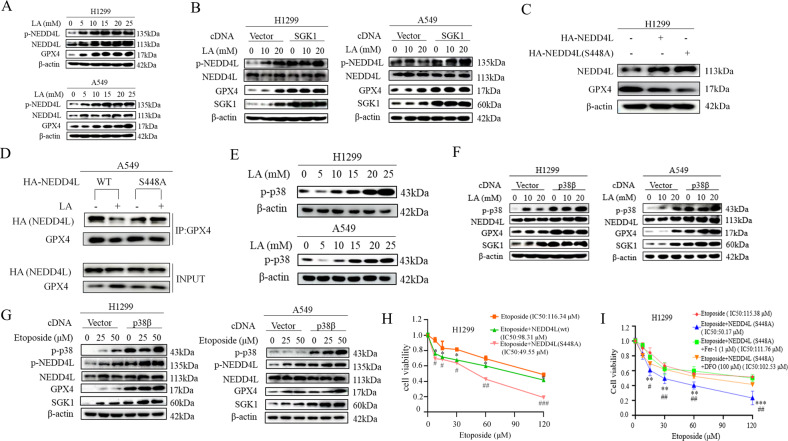
Activation of SGK1 by lactate reduced interaction of NEDD4L with GPX4. **A** A549 and H1299 cells were treated with lactate for the indicated concentrations, western blot was used to determine p-NEDD4L, NEDD4L, GPX4 protein levels. **B** After transfection with SGK1 cDNA for 36 h, A549 and H1299 cells were treated with lactate (0, 10, and 20 mM) for 3 h. Western blot was carried out for analysis of p-NEDD4L, NEDD4L, GPX4, SGK1 levels. **C** Cells were transfected with wild-type HA-NEDD4L cDNA or HA-NEDD4L (S448A) cDNA for 48 h, western blot was carried out for analysis of GPX4 levels. **D** After transfection with wild-type HA-NEDD4L cDNA or HA-NEDD4L (S448A) cDNA for 36 h, cells were untreated, or treated with lactate for 3 h. Cell lysates were immunoprecipitated with anti- GPX4 antibody and then Western blotted with anti- HA-NEDD4L. **E** Cells were treated with lactate for the indicated concentrations, Western blot was used to determine p-p38 protein levels. **F** After transfection with p38β cDNA for 48 h, A549 and H1299 cells were treated with lactate (0, 10 and 20 μM) for 3 h. Western blot was carried out for analysis of GPX4, NEDD4L, SGK1 levels. **G** After transfection with p38β cDNA for 36 h, A549 and H1299 cells were treated with Etoposide (0, 25 and 50 μM) for 12 h. Western blot was carried out for analysis of p-NEDD4L, NEDD4L, GPX4, SGK1 levels. **H** H1299 cells were treated with Etoposide, Etoposide plus NEDD4L (WT) cDNA and Etoposide plus NEDD4L (S448A) cDNA. Cell viability assay showed that NEDD4L cDNA significantly decreased H1299 cell viability. In H1299 cells, the IC 50 (half maximal inhibitory concentration) of etoposide was 19.87 μM, the cell survival was decreased in the presence of NEDD4L (WT) cDNA or NEDD4L (S448A) cDNA. (*p < 0.05, ^##^*p* < 0.01 for difference from etoposide-treated control by ANOVA with Dunnett’s correction for multiple comparisons). **I** H1299 cells were treated with Etoposide, Etoposide plus NEDD4L (S448A) cDNA in the presence and absence of Ferroptosis inhibitors Fer-1 or DFO. Cell viability assay showed that NEDD4L cDNA significantly decreased H1299 cell viability. Inhibition of ferroptosis with Fer-1 and DFO rescues S448A NEDD4L-increased sensitivity to etoposide. (**p < 0.01, for difference from etoposide-treated control, ^##^*p* < 0.01, for difference from etoposide plus NEDD4L (S448A) cDNA-treated control, by ANOVA with Dunnett’s correction for multiple comparisons).

In pursuit of the mechanism by which lactate regulated the binding of NEDD4L to GPX4, we were intrigued by the studies that found that NEDD4L is subjected to phosphorylation by serum- and glucocorticoid- inducible kinase 1 (SGK1) and such phosphorylation reduced the interaction between NEDD4L and its targets [11]. Because NEED4L phosphorylation was readily induced by either lactate or etoposide (Fig. 4A), we hypothesized that SGK1 might be an important mediator for lactate-dependent GPX4 regulation. We first tested this hypothesis by transfecting cells with SGK1 expression vector. Indeed, lactate-induced NEDD4L phosphorylation was significantly enhanced by ectopic expression of SGK1, remarkably, this was accompanied by a parallel rise of GPX4 levels (Fig. 4B). Since SGK1 phosphorylates NEDD4L at Ser 448, mutation of this residue from the serine to alanine should enhance NEDD4L interaction with GPX4, and resultant GPX4 ubiquitination and degradation. As expected, overexpression of wild-type NEDD4L decreased GPX4 levels in H1299 cells (Fig. 4C); importantly, GPX4 levels were further decreased by S448A mutant NEDD4L. Further support to the hypothesis was provided by immunoprecipitation experiments with anti-GPX4 antibody in H1299 cells transfected with either wild-type NEDD4L or mutant S448A NEDD4L. As shown in Fig. 4D, the interaction of NEDD4L with GPX4 was effectively reduced in wild-type-transfected cells exposed to lactate. In contrast, the reduction of the binding of NEDD4L to GPX4 by lactate was abolished in the cells transfected with S448A NEDD4L. These findings suggest that activation of SGK1 by lactate reduces association of NEDD4L to GPX4 and, consequently, dampens GPX4 degradation.

Next, we assessed SGK1 activation in response to lactate based on the recent reports that the expression of SGK1 is mediated mainly by p38 kinase. Indeed, lactate treatment increased the phosphorylation and activation of p38 kinase (Fig. 4E). Moreover, the levels of SGK1 and p-NEDD4L induced by lactate were markedly enhanced in p38β kinase-overexpressing cells, which was paralleled by an increase in GPX4 levels (Fig. 4F). similar results were observed in etoposide-exposed H1299 and A549 cells (Fig. 4G).

To assess the functional significance of NEDD4L-mediated degradation of GPX4, we examined whether NEDD4L-overexpressing cells increased sensitivity to etoposide. Strikingly, ectopic expression of wild-type NEDD4L increased etoposide sensitivity significantly, and that increase in sensitivity was further enhanced by transfection of cells with mutant S448A NEDD4L (Fig. 4H). Furthermore, inhibition of ferroptosis with Fer-1 and DFO rescues S448A NEDD4L-increased sensitivity to etoposide, indicating NEDD4L-mediated degradation of GPX4 is necessary for induction of ferroptosis and chemo-sensitivity (Fig. 4I).

### Mitochondrial ROS is required for lactate-dependent activation of p38-SGK1 pathway

**Fig. 5:**
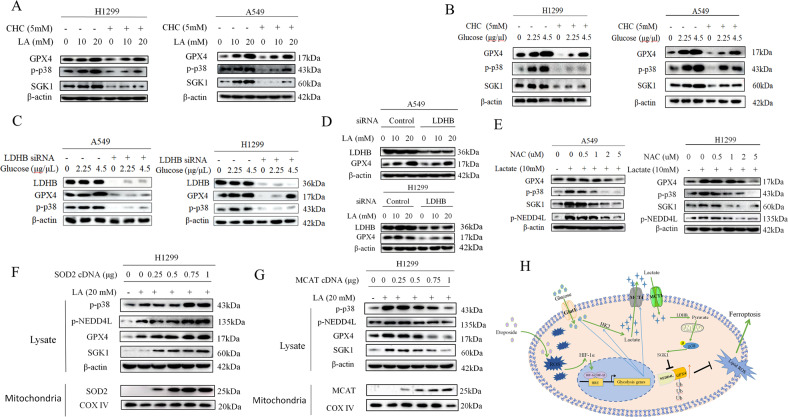
Mitochondrial ROS is required for lactate-dependent activation of p38-SGK1 pathway. **A** Western blot analysis of p-p38, SGK1 and GPX4 in A549 and H1299 cells treated with lactate in the presence of MCT1 inhibitor α-cyano-4-hydroxycinnamate (CHC). **B** Western blot analysis of indicated proteins in A549 and H1299 cells stimulated with glucose in the presence and absence of MCT1 inhibitor CHC. **C** After transfection with LDHB siRNA for 48 h, A549 and H1299 cells were treated with different doses of glucose for 3 h. Western blot was carried out for analysis of p-p38 and GPX4 levels. **D** After transfection with LDHB siRNA for 48 h, A549 and H1299 cells were treated with lactate (0, 10, and 20 μM) for 3 h. Western blot was carried out for analysis of GPX4 levels. **E** Western blotting shows expression levels after treatment with N-acetyl- l -cysteine (NAC) in the presence of 10 mM lactate. **F** 48 h after transfection with different doses of superoxide dismutase 2 (SOD2) expression plasmid, H1299 cells were transfected with 20 mM lactate for 3 h. Protein levels were prepared and analyzed by western blot with antibodies against indicated proteins. **G** 48 h after transfection with different concentrations of mitochondrial targeted catalase (MCAT) plasmid, H1299 cells were transfected with 20 mM lactate for 3 h. Western blot was used to detected the indicated proteins. **H** The chemotherapeutic drug etoposide induces metabolic reprogramming towards glycolysis in the NSCLC cells. The secreted lactic acid activates the expression of GPX4 protein to inhibit ferroptosis via p38/SGK1/NEDD4L pathway, thus contributing to chemoresistance in NSCLC.

The preceding data raise the issue of how lactate activated p38-SGK1 signaling axis. Recently, lactate has been identified as a key signaling molecule that has prominent roles in tumor progression. As we demonstrated in the recent study [21], lactate can be converted to pyruvate by lactate dehydrogenase B (LDHB) once it enters the cells, and go through oxidative phosphorylation (OXPHOS) in the mitochondria. The reactive oxygen species (ROS) resulting from the lactate-fueled OXPHOS drives activation of Akt pathway. Since p38 kinase is activated by various stress signals including ROS, this raised the possibility that lactate-induced ROS was responsible for p38 kinase activation. To test this, we first used monocarboxylate transport 1 (MCT1) inhibitor α-cyano-4-hydroxycinnamate (CHC) to block lactate transport into cells. CHC treatment caused the robust reduction of p-p38 kinase, as well as the GPX4 levels (Fig. 5A). To address the physiological relevance of activation of p38 and GPX4 expression induced by lactate, we evaluated whether modulation of glycolysis influences GPX4 expression. Both H1299 and A549 cells treated with glucose showed a significant increase p-p38 with a parallel rise of GPX4 (Fig. 5B), the addition of MCT1 inhibitor CHC led to decrease in the levels of p-p38 and GPX4 stimulated with glucose, indicating the critical role of glycolysis-derived lactate in activation of the p38 pathway as well as GPX4 expression. LDHB is a key player in the lactate-fueled OXPHOS cascade, notably, siRNA-mediated knockdown of LDHB similarly decreased levels of p-p38 kinase in tandem with the downregulation of GPX4 expression in the presence and absence of glucose (Fig. 5C). Similar results were obtained with treatment cells with lactate (Fig. 5D). To address the role of ROS induced by lactate-fueled OXPHOS in p38 activation, we then used the antioxidant N-acetyl-l-cysteine (NAC) to deplete the lactate-induced ROS levels. As shown in Fig.5E, p-p38 and p-NEDD4L levels were diminished and both GPX4 and SGK1 levels were inhibited in a dose-dependent manner by NAC treatment. To further determine whether lactate-induced mitochondrial ROS is functionally relevant for activation of p38 kinase, ROS scavenging enzyme was ectopically introduced into H1299 cells. Transfection with superoxide dismutase 2 (SOD2), which converts superoxide into hydrogen peroxide (H_2_O_2_) in mitochondria, strongly augmented lactate-induced p-p38 and p-NEDD4L levels (Fig. 5F). In stark contrast, transfection with mitochondrial targeted catalase (MCAT), which is targeted to mitochondria for detoxifying H_2_O_2_ [21], dose-dependently decreased the levels of p-p38 and p-NEDD4L (Fig. 5G). In these experiments, changes in p-p38 levels were consistently paralleled by changes in GPX4 and SGK levels (Figs. 5F, G). Our results collectively indicate that activation of p38/SGK1 axis by lactate-induced H_2_O_2_ is functionally relevant for GPX4 regulation.

## Discussion

The chemotherapeutic resistance is a major problem in cancer treatment. Ferroptosis is a new form of programmed cell death characterized by accumulation of lipid peroxides, which may provide a new opportunity for treating cancer. In this study, we demonstrated the chemotherapeutic agent etoposide can induce ferroptosis in lung cancer cells in a GPX4-dependent manner. Furthermore, our data showed that etoposide controls ferroptosis sensitivity through its ability to regulate metabolic reprogramming. Our in vitro model using Fer-1, DFO, and knockdown of GPX4 indicated the involvement of ferroptosis in etoposide-induced cell death in SCLC. However, in NSCLC Cells, etoposide rewires the metabolic programming to upregulate the GPX4 for suppressing ferroptosis. The main mechanism of ferroptosis resistance in NSCLC relies on the lactate accumulation in tumor microenvironment resulting from the metabolic reprogramming. Lactate increases mitochondrial ROS generation and drives activation of p38-SGK1 pathway, which attenuates the interaction of NEDD4L with GPX4 and subsequent ubiquitination and degradation of GPX4. Hence, our findings reveal a novel mechanism by which tumor cells exploit metabolic reprogramming to induce resistance to ferroptosis and chemotherapy.

Emerging evidence has confirmed that different types of cancer show varying sensitivities to ferroptosis [4]. Our results found etoposide promotes metabolic reprogramming to suppress ferroptosis in NSCLC cells rather than in SCLC. The etoposide-enhanced glycolysis is manifested by upregulation of glycolysis gene expression, resulting in increased glucose uptake and lactate production. But why does metabolic reprogramming occur in NSCLC? It has been reported that under basal conditions, NSCLC have lower ROS levels than SCLC due to higher glutathione (GSH) levels [22, 23]. We speculate that ROS levels in SCLC cells are already too high to be further elevated compared to those in NSCLC cells. Another potential mechanism to consider is the likely slow kinetics of metabolic rewiring associated with chemotherapy resistance in SCLC. Importantly, accumulated lactate promotes GPX4 upregulation and mediates ferroptosis resistance in NSCLC cells. Our study clearly showed the survival of etoposide-resistant NSCLC cells could not be explained by genetic mutation mechanisms; rather, the adaptive responses are associated with increased resistance to the drug treatment. These results also support the view that, in recent years, lactate is no longer a metabolic waste, but is involved in the development of tumors [17].

So far, there is no specific marker to characterize ferroptosis, but abundant evidence demonstrates that glutathione peroxidase 4 expression controls the initiation of ferroptosis [6]. GPX4 is an important kinase in maintaining intracellular oxidation balance, especially in catalyzing the formation of non-toxic alcohols from lipid peroxides [24]. In lung cancer cells, manipulation of GPX4 expression changed the content of corresponding markers and cell viability, suggesting that GPX4 controls the occurrence of ferroptosis. Importantly, our results show that etoposide upregulates GPX4 expression in NSCLC cells, suggesting that GPX4 may influence etoposide resistance in lung cancer cells.

We also demonstrated that lactate regulates GPX4 expression through post-transcriptional ubiquitination. In the past, studies on post-transcriptional regulation of GPX4 were rare. It has been reported that the selenium response element on GPX4 mRNA is critical to the protein translation process [25]. Heat shock protein HSPA5 acts as a molecular chaperone and interacts with GPX4 to improve its intracellular stability [26]. However, the E3 ubiquitin ligase of GPX4 has not been reported, and the molecular mechanism of ubiquitin regulation of GPX4 is still unknown. We demonstrated for the first time that NEDD4L, neural precursor cell expressed developmental down-regulated protein, is the E3 ubiquitin ligase of GPX4 and specifically mediates the regulation of GPX4 by lactate. Remarkably, at the same time, our evidence showed that NEDD4L inactivation was mediated by mitochondrial ROS-activated p38MAPK-SGK1 pathway.

Taken together, we propose a new drug resistance model in NSCLC cells, where etoposide induces cell metabolic reprogramming, leading to the accumulation of lactic acid in the microenvironment; Lactic acid inhibits GPX4 ubiquitination and promotes GPX4 accumulation by inactivating the E3 ubiquitin ligase NEDD4L, resulting in ferroptosis resistance in NSCLC. Our findings position a key role of metabolism rewiring associated with lactate production in drug resistance of NSCLC. Since increased lactate is also linked with tumor metastasis and poor prognosis, lactate metabolism can be exploited for therapeutic targeting.

Finally, having established the central role of GPX4 in dictating sensitivity to ferroptosis induction in NSCLC opens up many therapeutic possibilities. Targeting GPX4 could re-sensitize drug-resistant NSCLC to chemotherapies. Conversely, boosting ferroptosis, such as inhibition of SLC7A11 or augmenting lipid peroxidation, could strengthen chemotherapeutic efficacy in NSCLC. Collectively, our findings will provide new ideas for the treatment of NSCLC and add new targets for combination therapy.

## Materials and methods

### Plasmid and short interfering RNA transfection

Cells seeded in six-well plates were grown to 70% confluence before plasmids transfection and it was done with PolyJet DNA Transfection Reagent (Signa-Gen Laboratories, Gaithersburg, MD, USA) according to the manufacturer’s instructions. The transfection with siRNA was performed by using GenMute siRNA Transfection Reagent (Signa-Gen Laboratories) when cells seeded in plates were grown to 30–50% confluence. All the siRNAs were purchased from GENERAL BIOSYSTEMS (Chuzhou, China). After transfection for 36 h, cells were deprived of serum and growth factors for 12 h and then treated with lactic acid (Roche, San Francisco, CA, USA) for 3 h and harvested. The sequences of the siRNAs are listed in Table 1.

**Table 1:** Sequence of siRNA.

Gene	Genebank accession number	Target sequence (5′–3′)
siLDHA	NM_001165414.1	GCCAUCAGUAUCUUAAUGATT
siLDHB	NM_002300.8	AAGAUUGUAGUGGUAACUGCATT
siHIF1a	NM_001530	CCAGCAGACUCAAAUACAATT
siGPX4	NM_002085.5	UGGUGAUAGAGAAGGACCUTT
siNEDD4L	NM_001144967.3	GGUCCUCAGCUGUUUACAA

### Supporting materials and methods

Cells, transfection, antibodies, plasmids, reagents, cloning, and DNA construction and extended experimental procedures can be found in Data S1. The full length bands of WB can be found in Data S2.

## Supplementary information

Data S1

Data S2

## Data Availability

The data used or analyzed in this study are available from the corresponding author upon reasonable request.
