# Supplementary material for: Drug-induced lactate confers ferroptosis resistance via p38-SGK1-NEDD4L-dependent upregulation of GPX4 in NSCLC cells
Source: Cell Death Discov. 2023 May 15;9:165. doi: 10.1038/s41420-023-01463-5 (PMC10185500; doi:10.1038/s41420-023-01463-5)
Supplement: Supplementary file 1 — Data S1 [file 41420_2023_1463_MOESM1_ESM.docx]

**Supporting Materials and Methods**

**Cell culture, antibodies and reagents**

H1299 (non-small cell lung cancer), A549 (non-small cell lung cancer), H446 (small cell lung cancer) and H1688 (small cell lung cancer) cell lines were cultured with DMEM (GibcoThermo Fisher Scientific, Waltham, MA, USA). Anti-Ubiquitin (3933s), anti-LDHA (3582s) and anti-Phospho-p38 (9211s) were obtained from Cell Signaling Technology. Anti-SLC16A4 (MCT4) (A3016), anti-GLUT1 (A6982), anti-SGK1 (A3936), anti-NEDD4L (A9078), and anti-Phospho-NEDD4L (AP0843) were obtained from Abclonal (Wuhan, China). Anti-β-Actin (A1978) was obtained from Sigma-Aldrich (St. Louis, USA). Anti-GPX4 (ab125066) was purchased from Abcam (Cambridge, UK). Anti-HK2 (K001797P), anti-LDHB (K109376P) and anti-COX IV (K000016M) were obtained from Solarbio (Beijing, China). Anti-MCT1 (sc-365,501) were obtained from Santa Cruz Biotechnology (Santa Cruz, CA, USA). Anti-Mn SOD (SOD2) from Stress Gene (Victoria, BC). Anti-human catalase (MCAT) from Athens Research Technology (Athens, GA). Ferstatin-1, DFO, Erastin and Z-VAD-FMK were obtained from MCE (New Jersey, USA). Etoposide solution (H20143143) was obtained from QILU PHARMACEUTICAL Co.,Ltd. (Hainan, China). Cycloheximide (no.239763) were purchased from Sigma (Sigma-Aldrich Co., St Louis, MO, USA).

**MTT Assay**

Cell viability was determined using a 3-(4,5-dimethylthiazol2-yl)-2,5-diphenyl tetrazolium bromide (MTT) (Beyotime Institute of Biotechnology, Shanghai, China) assay. After indicated treatment, cells were washed with PBS and incubated with fresh medium with MTT solution (0.5mg/mL) for 4h at 37°C, the resulting formazan crystals were dissolved in DMSO and absorbance at wavelength of 570 nm was taken on a plate reader using BioTek citation 5 (BioTek, Winooski, VT, USA).

**ROS Assay**

Cells (2 × 10^5^) in 6-well plate were treated with etoposide for 10 h. Then the reactive oxygen species (ROS) fluorescence intensity was measured using Reactive Oxygen Species Assay Kit (Beyotime, Shanghai, China) by a fluorescence microplate reader Software Gen 5 (BioTek, Winooski, VT, USA).

**Lipid ROS Assay**

Indicated cells were seed in 6-well plate, then treated with etoposide for 12h. Add 5µM C11-BODIPY to cell culture medium for 30min at 37°C.Cells were harvested by trypsinization and resuspended in 500µL of cold PBS, then strained through a 40 µm cell strainer. Cells were subjected to flow cytometry analysis equipped with a 488 nm laser for excitation. Data were collected from the FL1 channel. A minimum of 10,000 cells were analyzed per condition. The data analysis was performed by using FlowJo Version 7.6 software.

**Western Blot**

Cells were treated and homogenized using heated laemmli sample buffer (S3401, Sigma, Victoria, BC, Canada). The homogenate was separated by SDS-PAGE and transferred to nitrocellulose (NC) membrane (GE Healthcare, Piscataway, NJ, USA). After detecting with various antibodies, signal was scanned by Chemiluminescence system AI680 (Protein Simple, San Jose, CA, USA).

**Dual luciferase reporter assays**

The GPX4 reporter constructs were co-transfected with Renilla luciferase vector into the cells. The expression levels of firefly luciferase and Renilla luciferase were determined by dual-luciferase reporter assay system following the manufacturer’s protocol (Promega). All the results are presented as average value of triplicates ± SD.

**Quantitative real-time RT–PCR analysis**

Total RNA was prepared from the various treated cells using TRIzol (Invitrogen) according to the manufacturer’s instructions. For real-time quantitative RT-PCR (qRT-PCR) analyses the cDNA was synthesized using PrimeScript First Strand cDNA Synthesis Kit (TaKaRa Bio, DaLian, China), and mixed with TB Green Master Mix (Applied Biosystems, Thermo Fisher Scientific, Waltham, MA, USA). The amplified products were measured using Roche LightCycler 96 (Roche). Each sample was repeated three times and analyzed using relative quantification software (Applied Biosystems).The primers used were:

GPX4forward:5’-GCACATGGTTAACCTGGACA-3’ GPX4 reverse:5’-CTGCTTCCCGAACTGGTTAC-3’

Beta-actin forward:5’-CCTTCCTG GGCATGGAGTCCT-3’

Beta-actin reverse:5’- GGAGCAATGATCTTGATCTTC-3’

**Calculation of detailed ratio in total cell death after Etoposide treatment**

We showed an example of calculations with data sets of SCLC H446 cell line after Etoposide treatment. Ferroptosis is defined as the percentage of cell death rescued by Fer-1 in Etoposide-induced cell death (70.5-59%=11.50%). Apoptosis is defined as the percentage of cell death rescued by z-vad-fmk in Etoposide-induced cell death (70.05-59%=11.05%). Total % (100%) are divided into two categories in accordance with their ratios (11.50:11.05).

The detailed calculations are shown below.

Average percentage of cell survival is as follows.

A:100% in Etoposide (-), Fer-1 (-), z-vad-fmk (-) group

B: 59% in Etoposide (+), Fer-1 (-), z-vad-fmk (-) group

C: 70.50% in Etoposide (+), Fer-1 (+), z-vad-fmk (-) group

D: 70.05% in Etoposide (+), Fer-1 (-), z-vad-fmk (+) group

We defined the percentage of ferroptosis: C-B=11.50%

We defined the percentage of apoptosis: D-B=11.05%

If we assume that Etoposide-induced cell death consisted of ferroptosis and apoptosis, the total % (100%) was divided into two categories (ferroptosis and apoptosis) in accordance with their ratios (11.50:11.05)

Ferroptosis=100*[11.50/(11.50+11.05)]=51%

Apoptosis=100*[11.05/(11.50+11.05)]=49%

**Cloning and DNA construction**

The GPX4 promoter reporters were constructed by inserting the amplified promoter fragments into the luciferase reporter vector PGL3-basic (Promega, Madison, WI, USA) at the Kpn I and Hind III sites.

The primers used were:

GPX4 forward: 5’-CGGGGTACCCGGGAGCAAAGCCATGTGAACAGA-3’

GPX4 reverse: 5’-CCCAAGCTTTCCAACTCGCAGAAAAGTGTCCCC-3’

**Immunoprecipitation (IP)**

The cells were collected in an IP lysis buffer containing the protease inhibitor PMSF (beyotime, ST505) and 1 x protease inhibitor cocktail (Roche). The same amount of lysate per sample was incubated with GPX4 antibody overnight at 4° with IgG as a control. The mixture was further incubated with Protein A/G Agarose (Sigma, Victoria, BC, Canada) for 5 h, and washed with cold PBS buffer. 60 μl of loading buffer containing loading buffer was then added, followed by boiling at 100 ° C for 10 minutes. Protein expression analysis was performed by Western blotting.

**Mitochondrial separation**

The SOD2 or MCAT cDNA were transfected in H1299 cells, and mitochondrial separation was performed using the cell mitochondrial isolation kit (Beyotime; C3601) according to manufacturer’s protocol. The level of the relevant proteins was detected by Western blotting.

**Lactate and glucose uptake determination**

Cells (2 × 10^5^) were treated with Etoposide for 10 h. Lactate in the Culture medium was measured using the Lactate Assay Kit (BioVision, Milpitas, CA, USA) according to the manufacturer’s instructions. The concentration of lactate was determined using Lactate Standard Curve.

**Statistical analysis**

Statistical analyses were performed with analysis of variance (ANOVA) using GraphPad Prism 6 (GraphPad Software Inc., San Diego, CA, USA) and are presented as mean ± S.D. from triplicated independent experiments. A significant difference was considered when the P-value from a two-tailed test was <0.05.
