# Supplementary material for: Drug-induced lactate confers ferroptosis resistance via p38-SGK1-NEDD4L-dependent upregulation of GPX4 in NSCLC cells
Source: Cell Death Discov. 2023 May 15;9:165. doi: 10.1038/s41420-023-01463-5 (PMC10185500; doi:10.1038/s41420-023-01463-5)
Supplement: Supplementary file 2 — Data S2 [file 41420_2023_1463_MOESM2_ESM.docx]

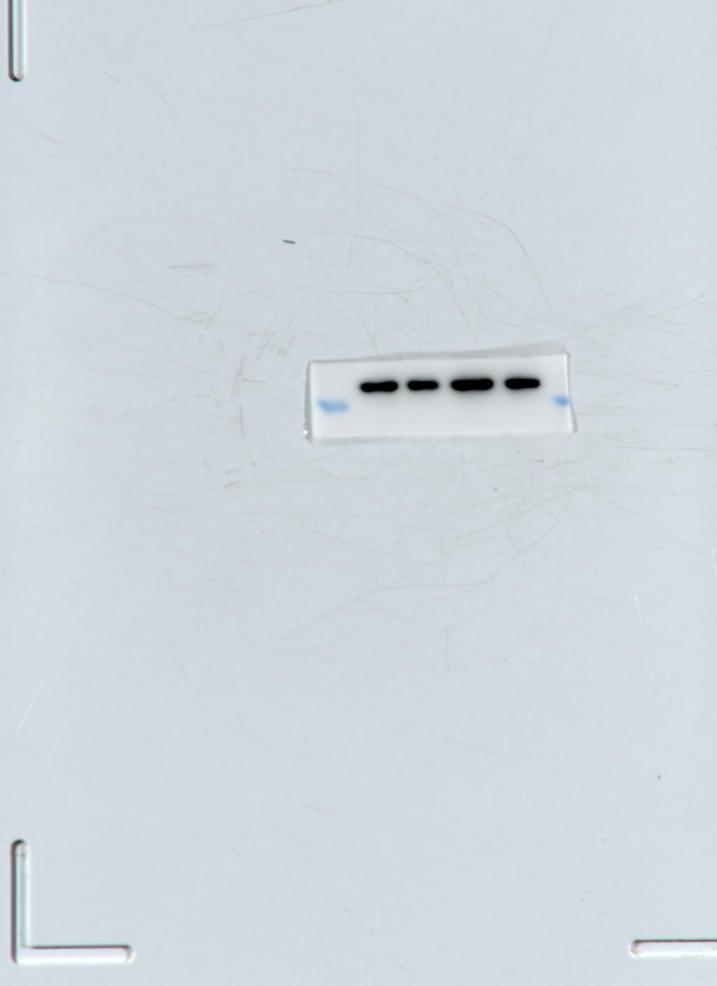


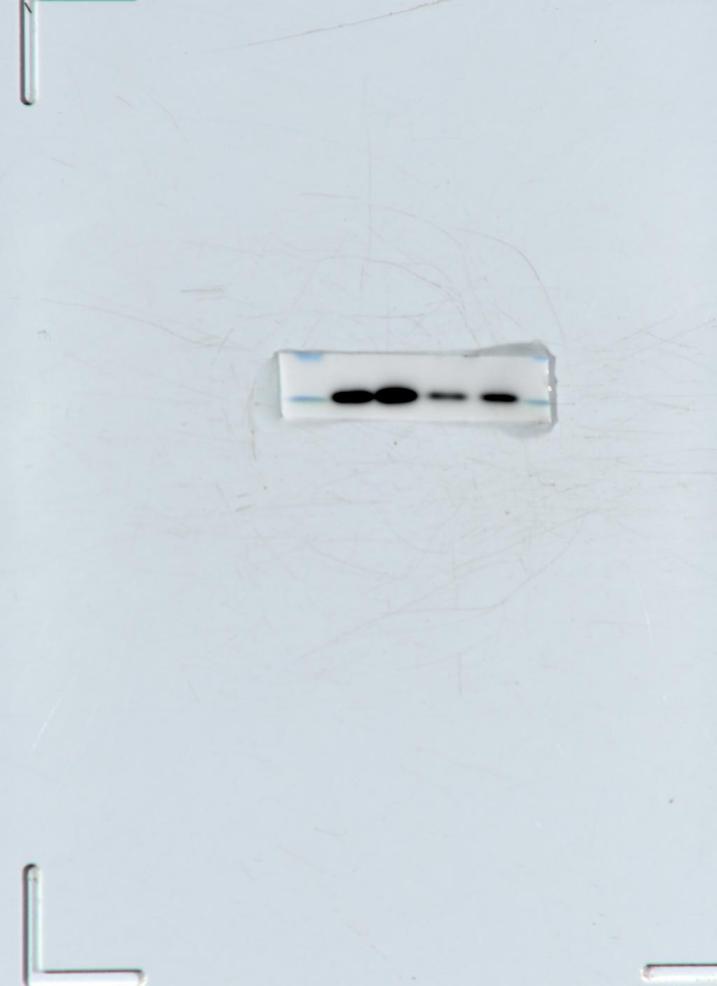
FIGURE 1E


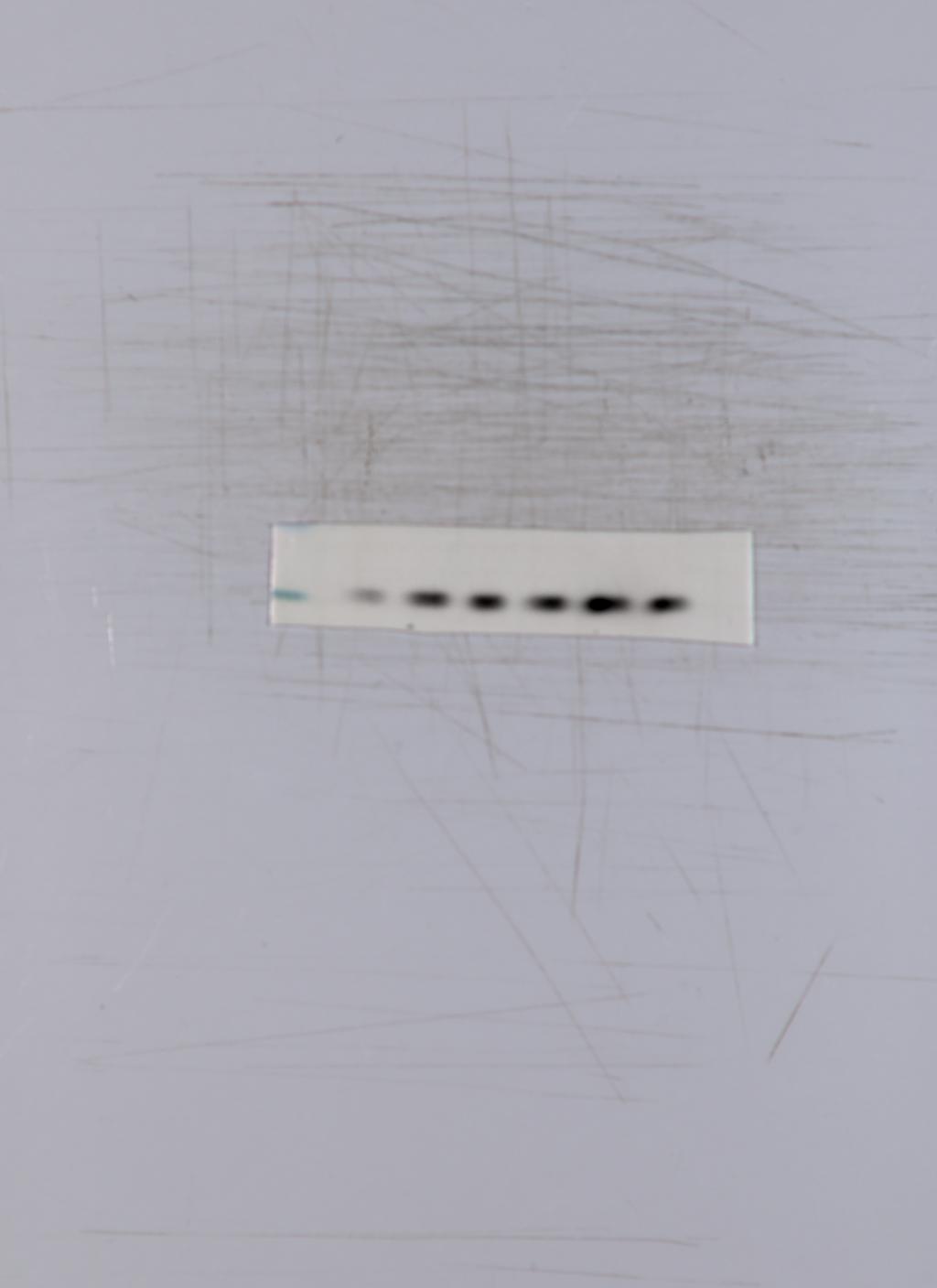

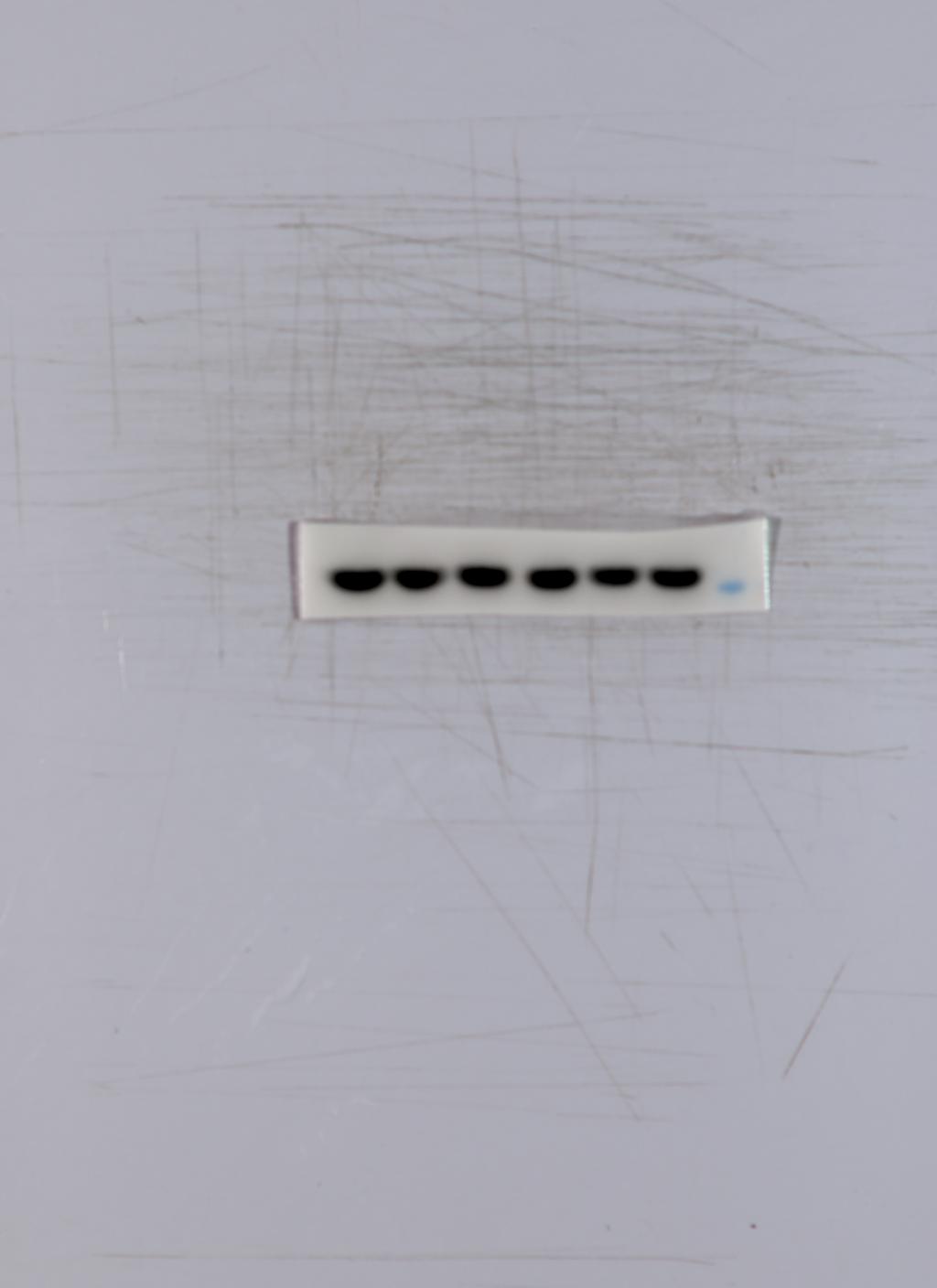

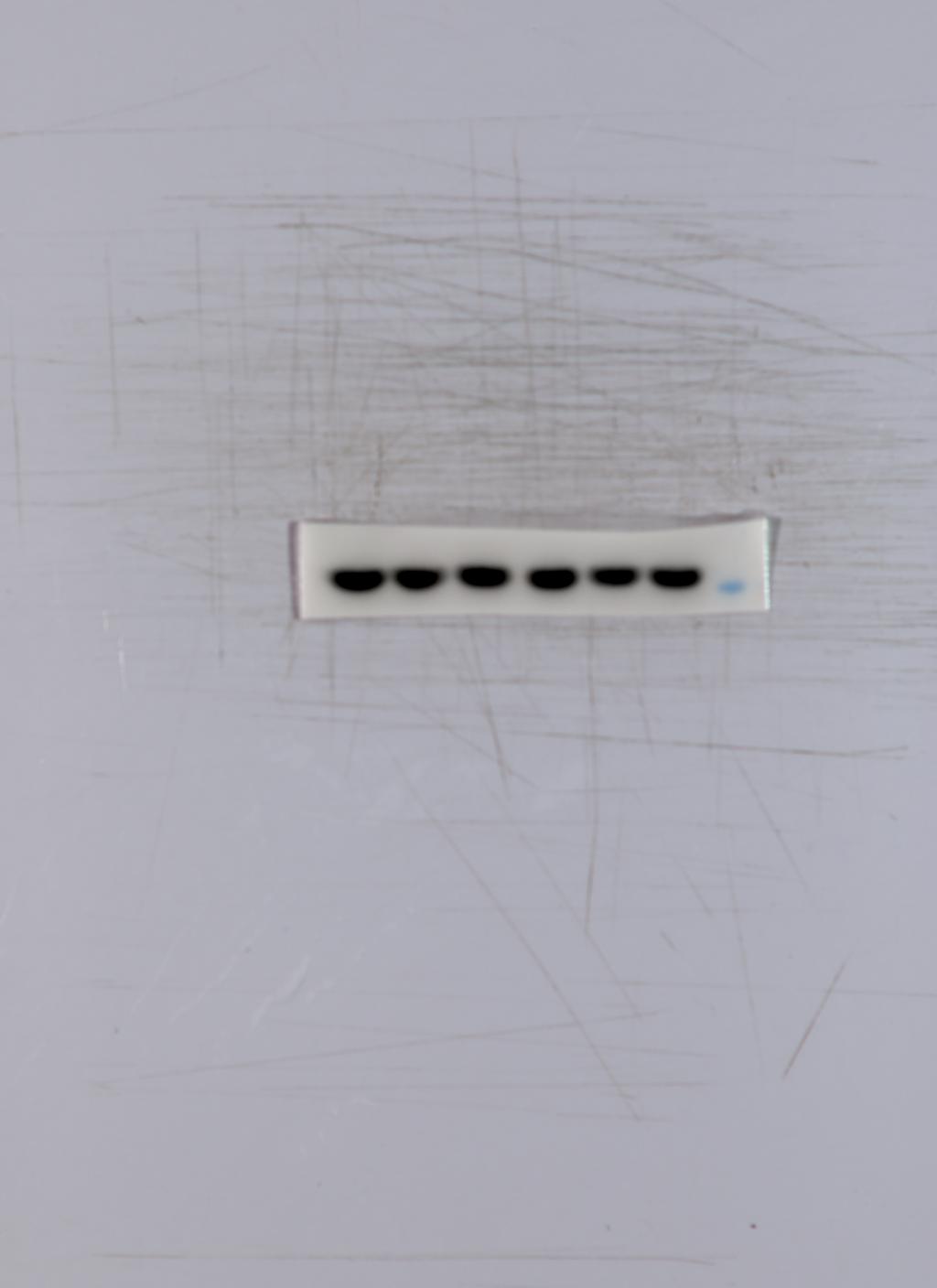

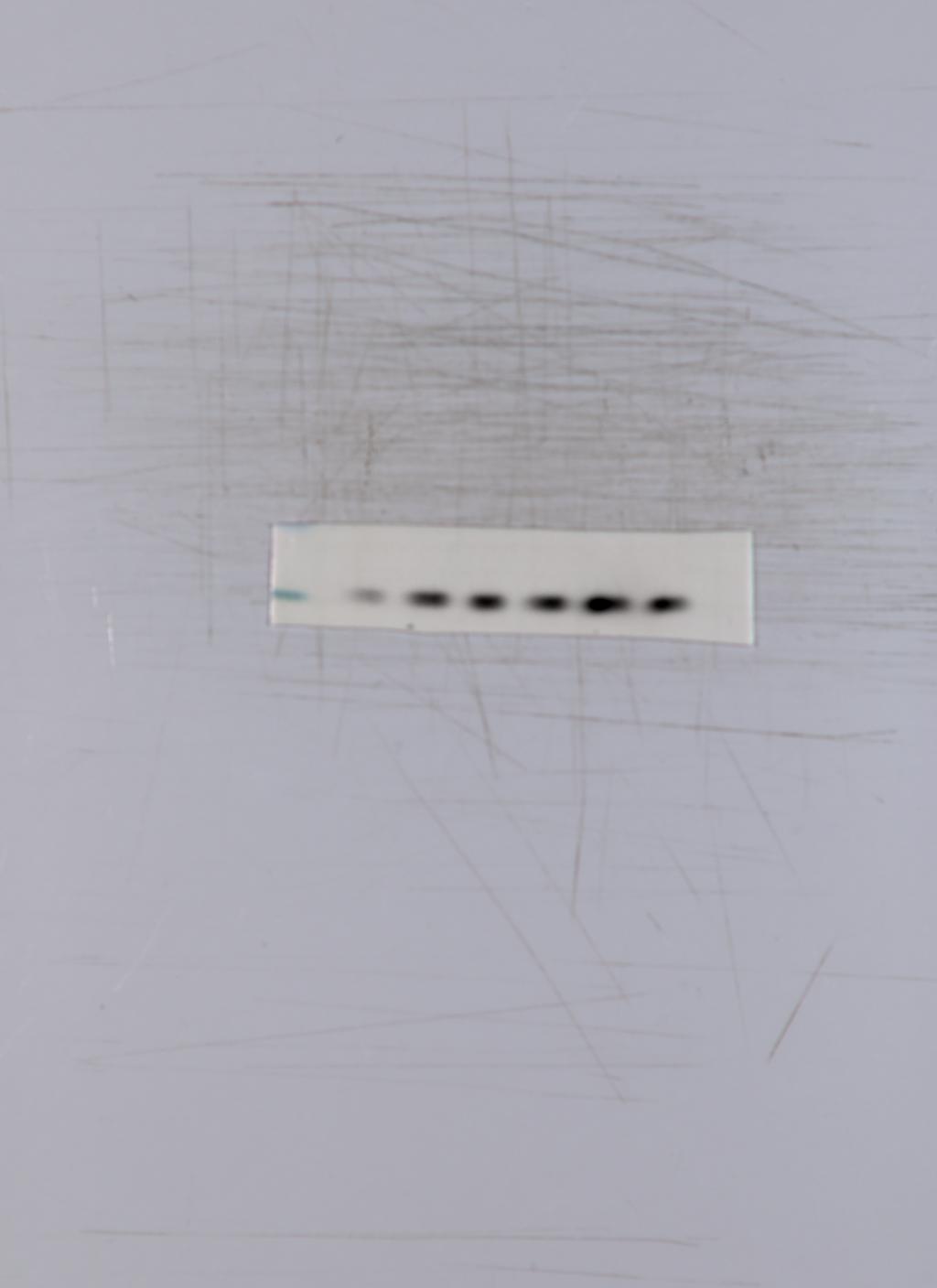


FIGURE 1I


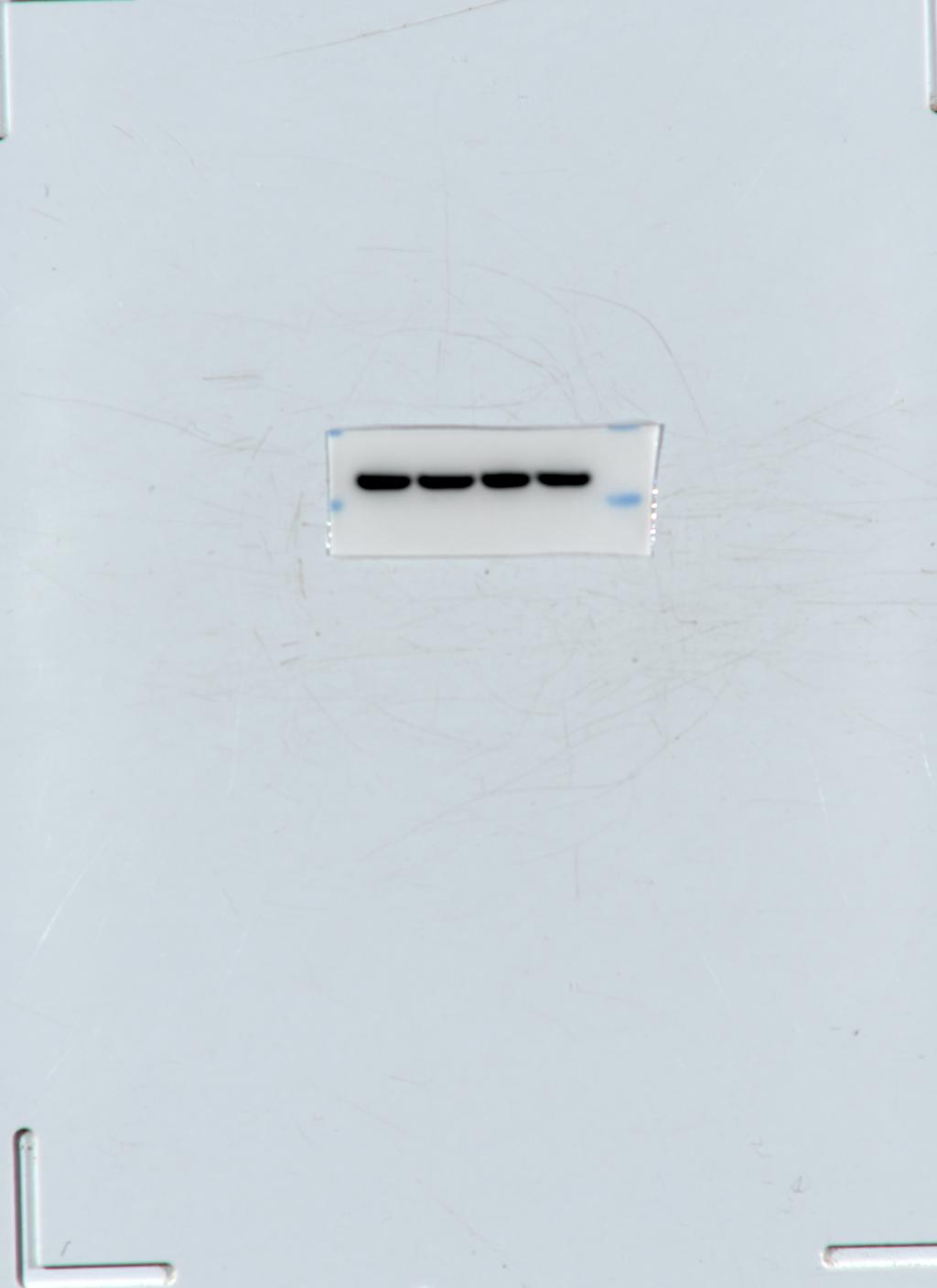


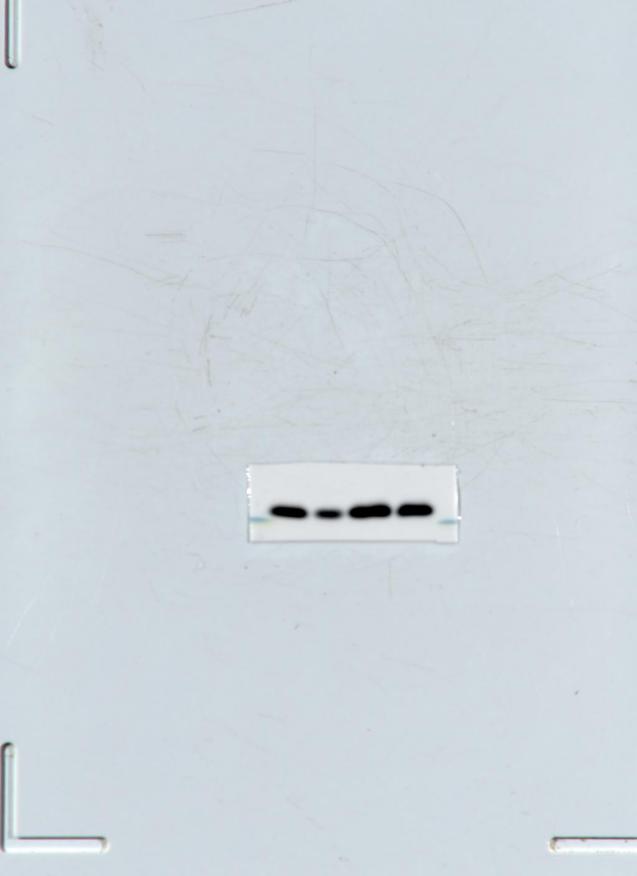


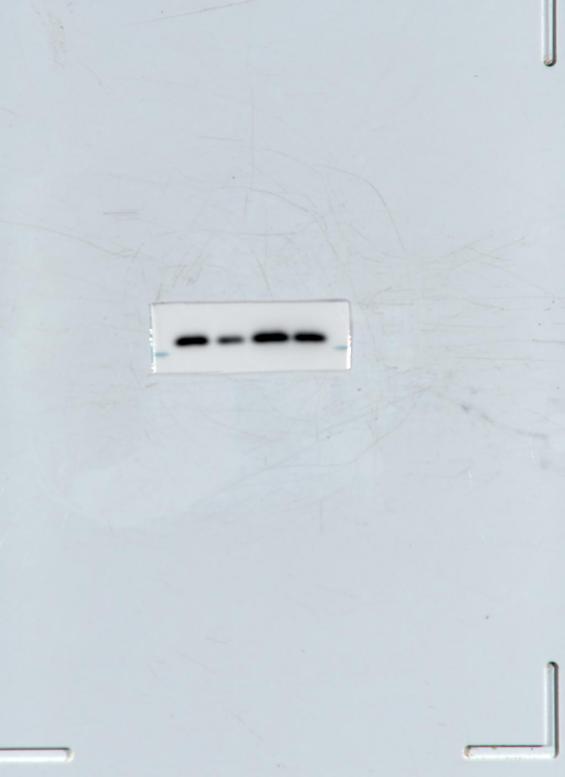

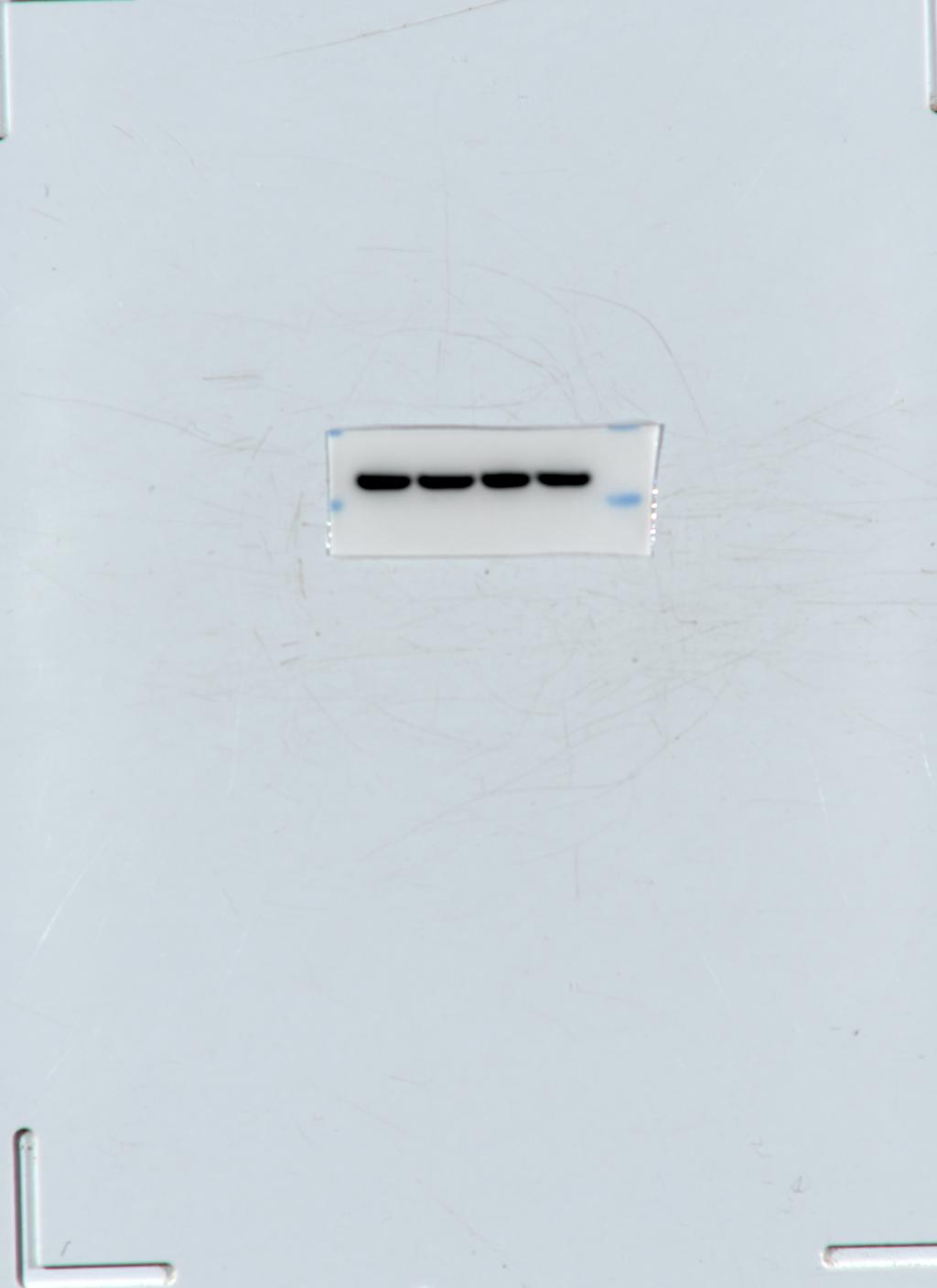


FIGURE 1J


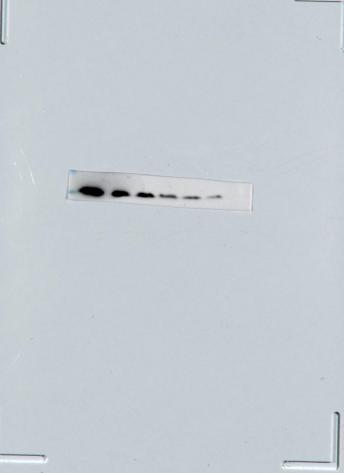

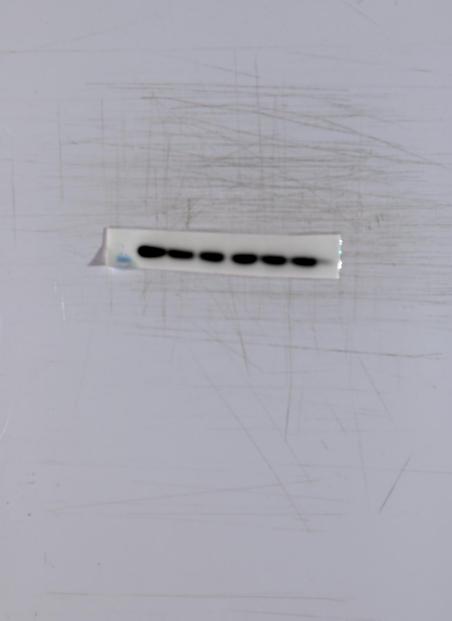


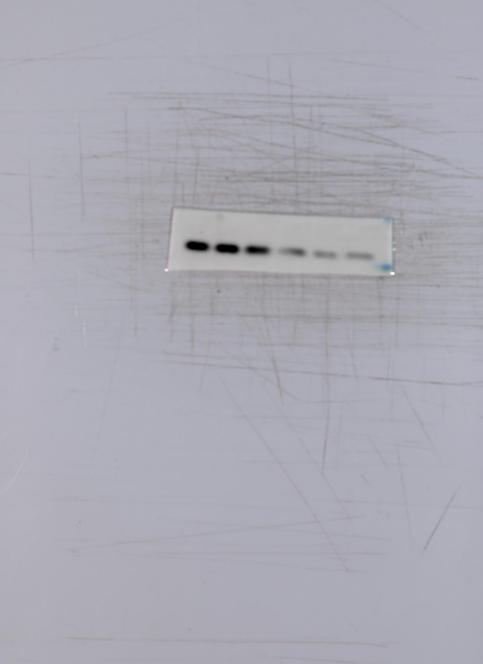


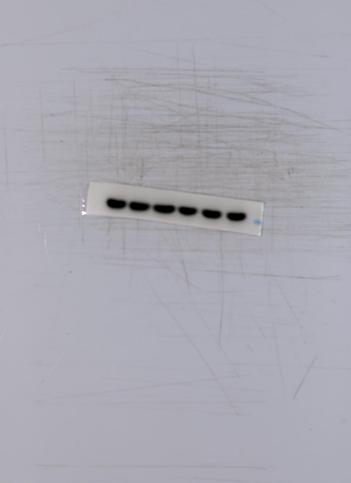


FIGURE 1K


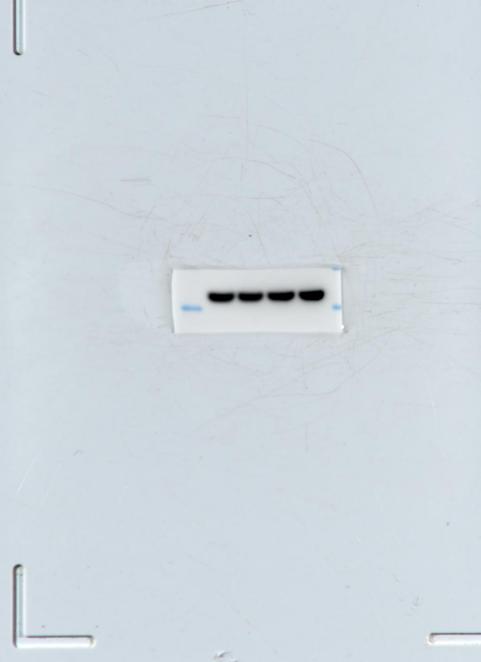


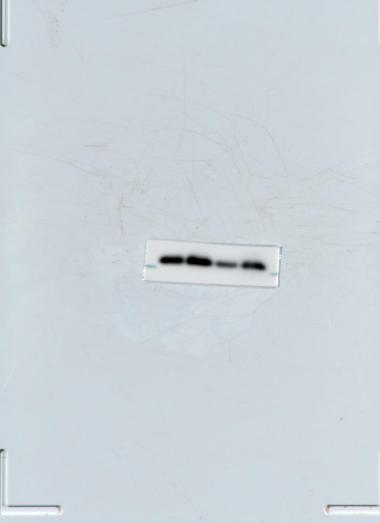


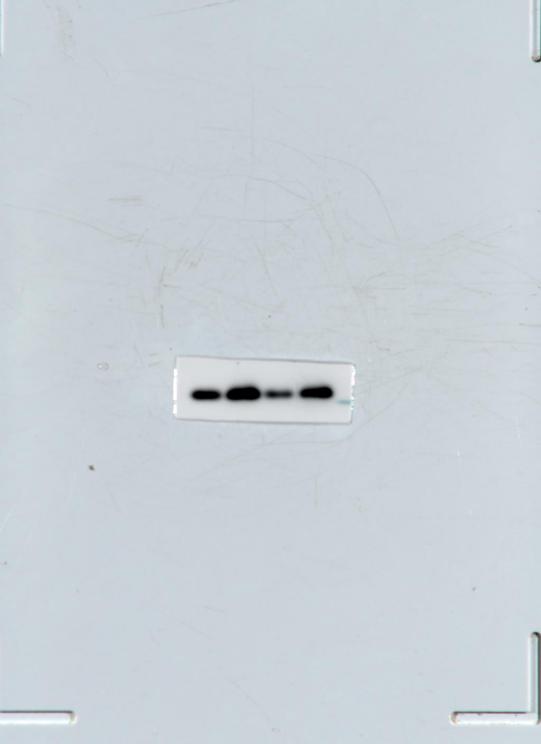


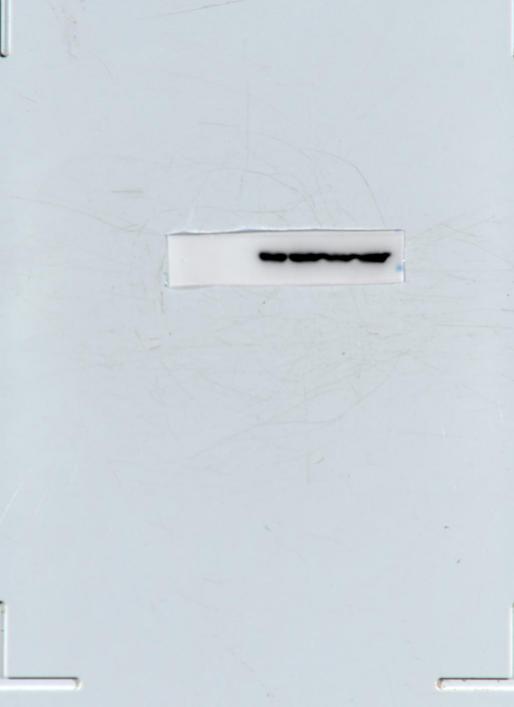


FIGURE 1L


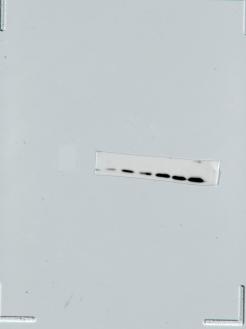


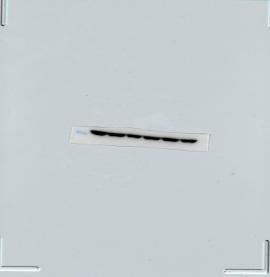

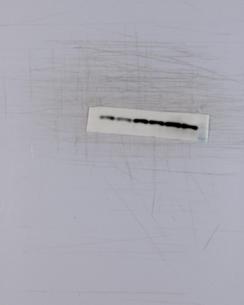


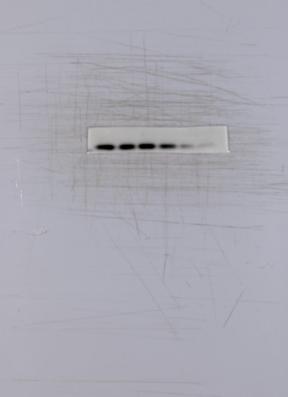

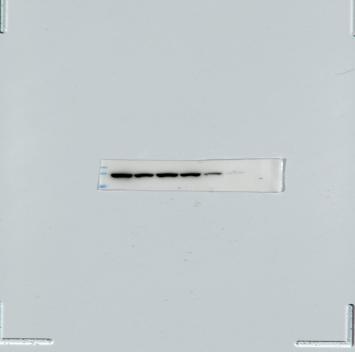

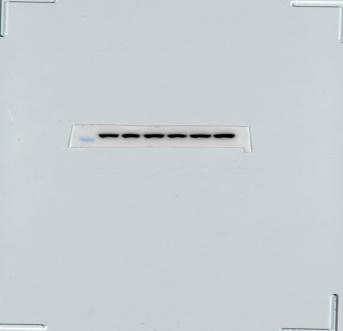


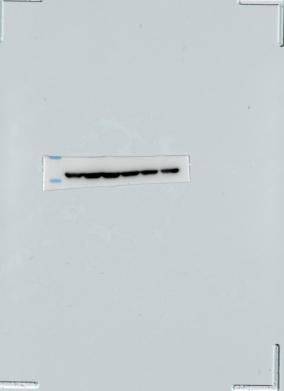


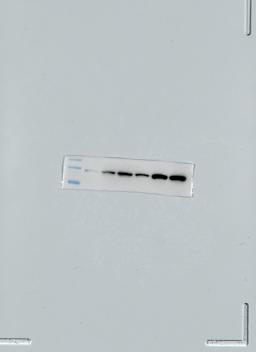


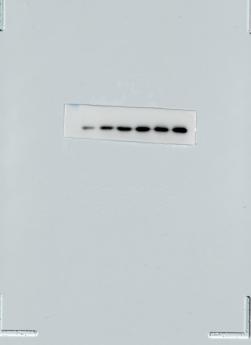


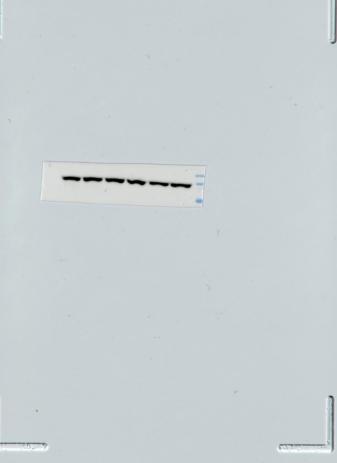


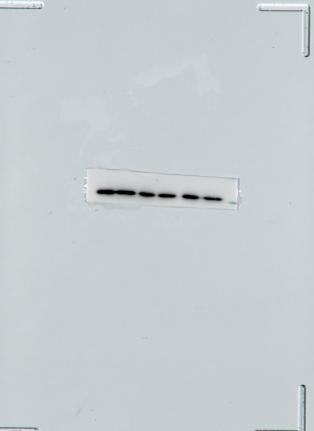

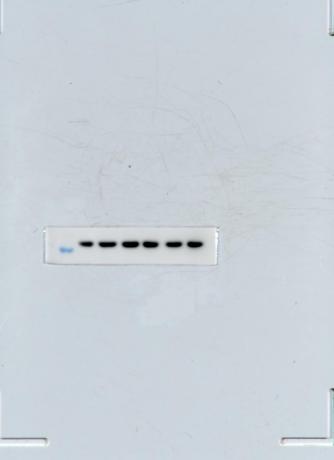


FIGUR 2A


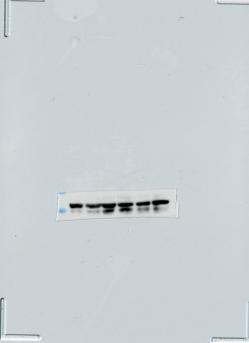

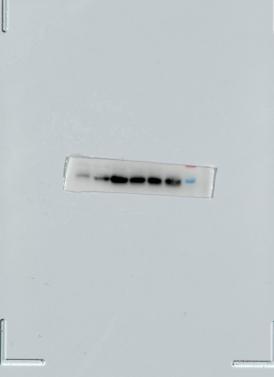


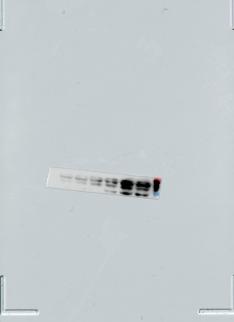

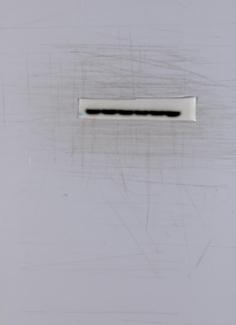


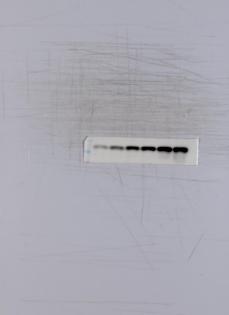


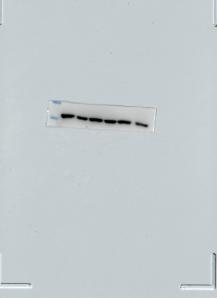

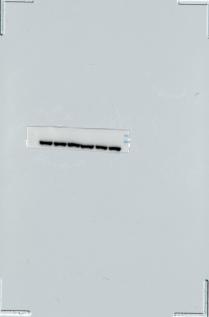

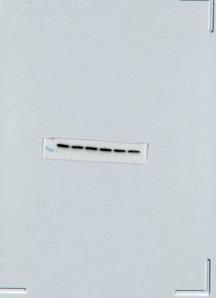

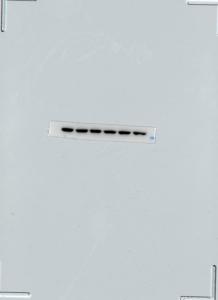


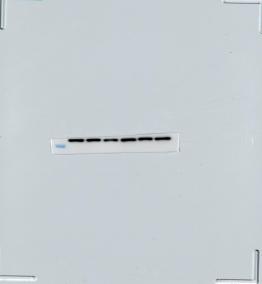


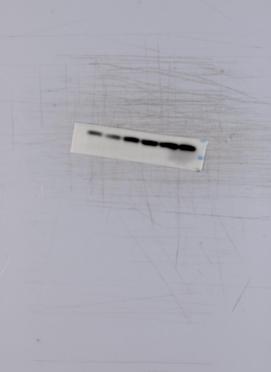

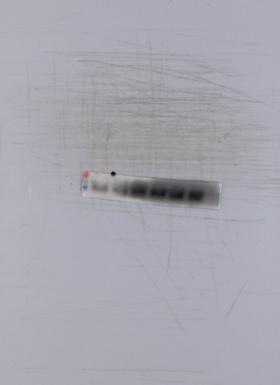


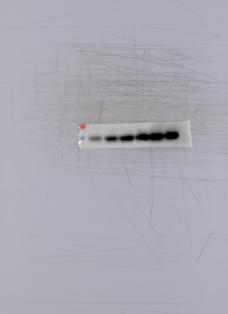


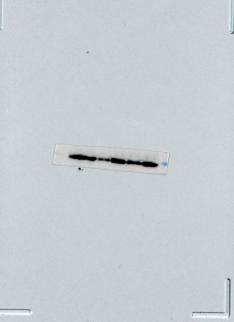

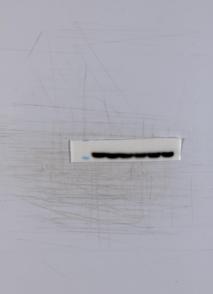


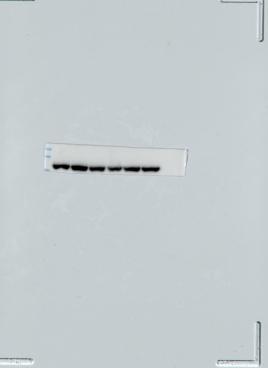

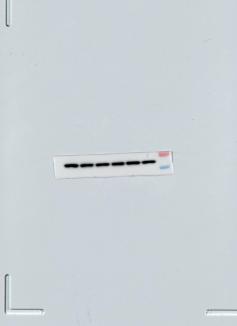

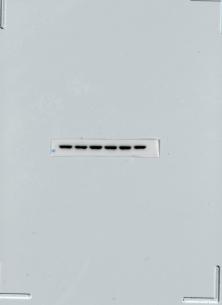


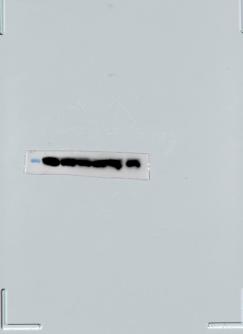

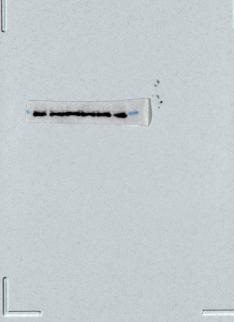


FIGURE 2C


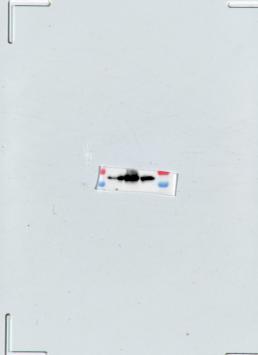

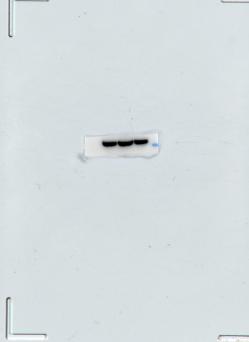

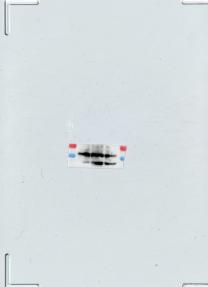


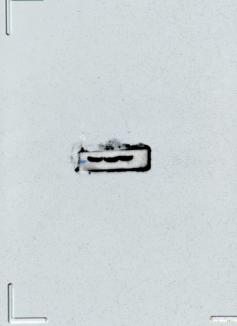


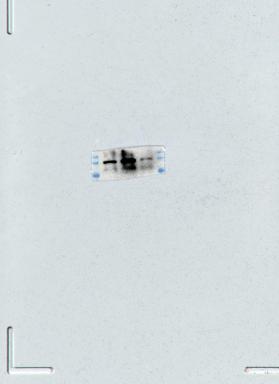


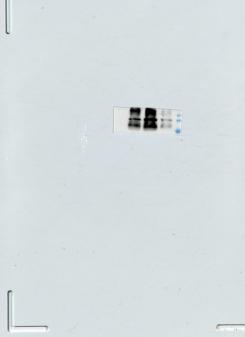


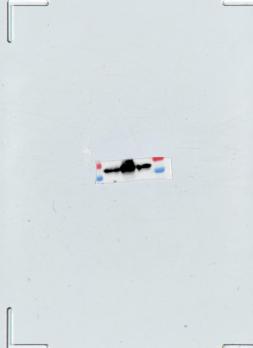

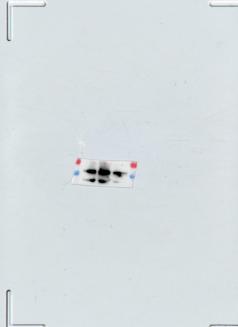


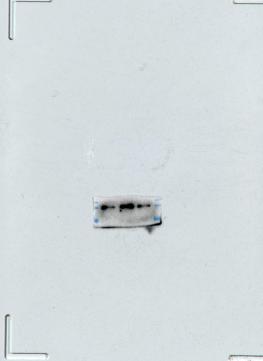


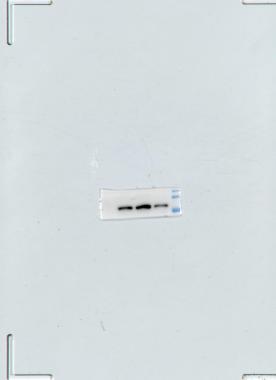


FIGUE 2D


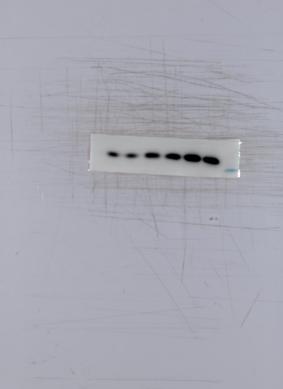

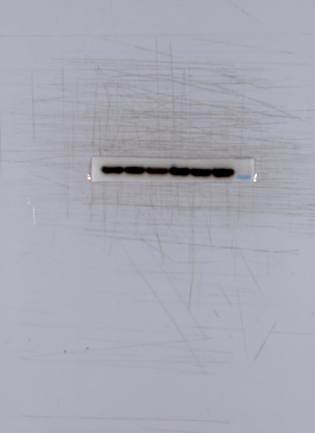


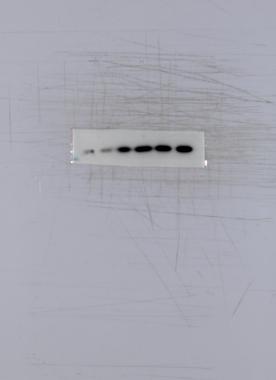


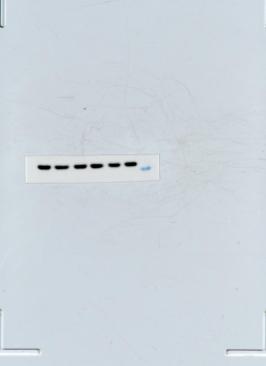


FIGURE 2G


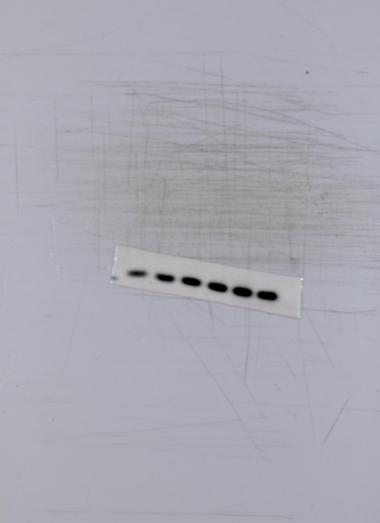

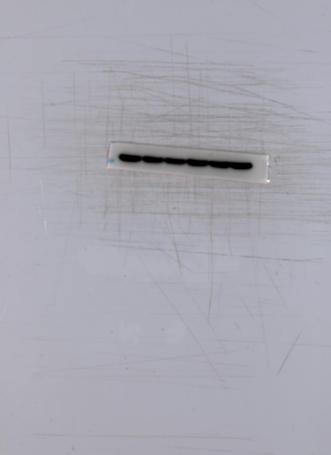


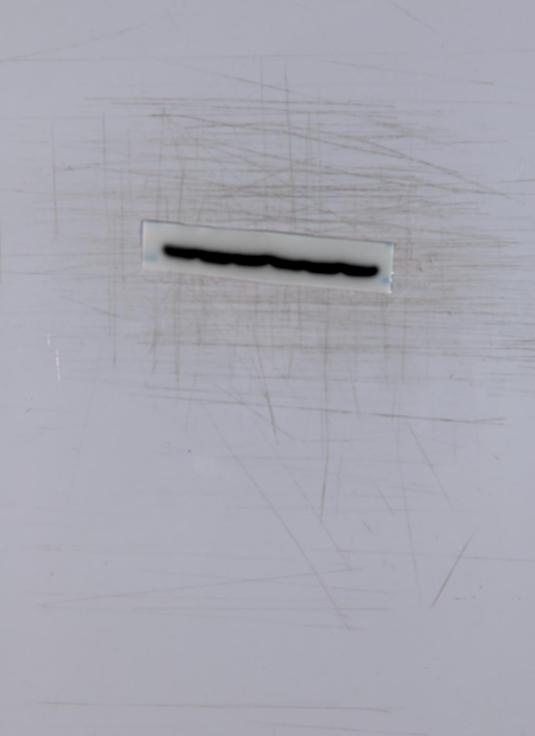


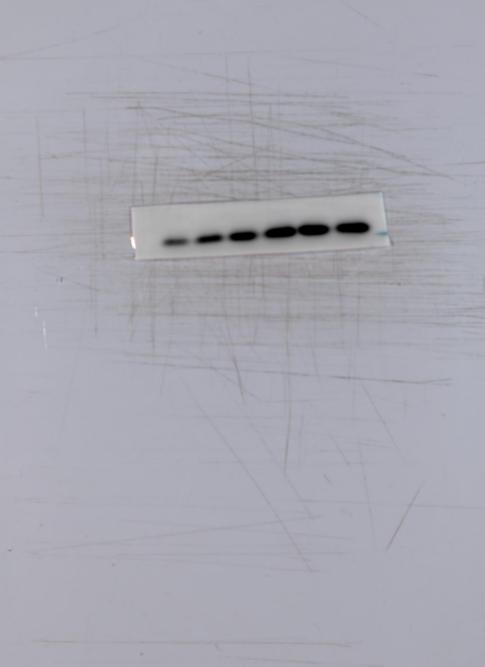


FIGURE 2H


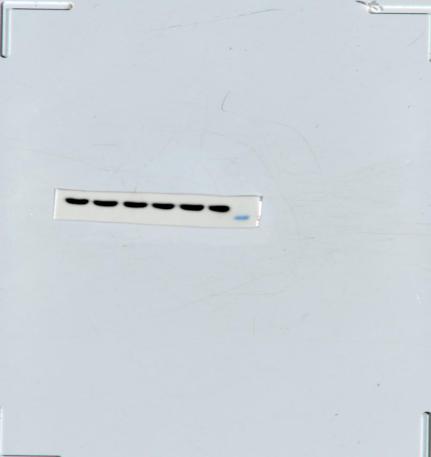

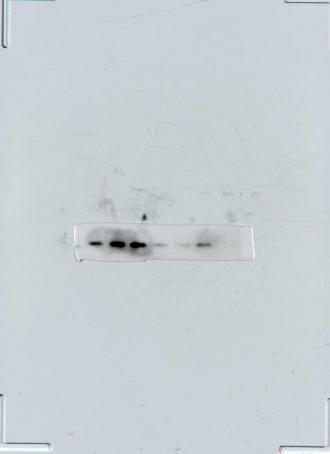


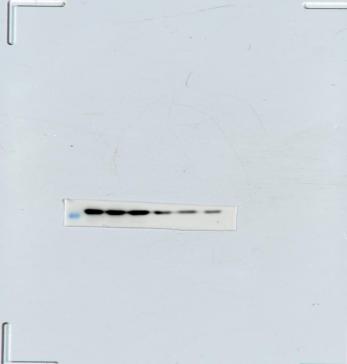


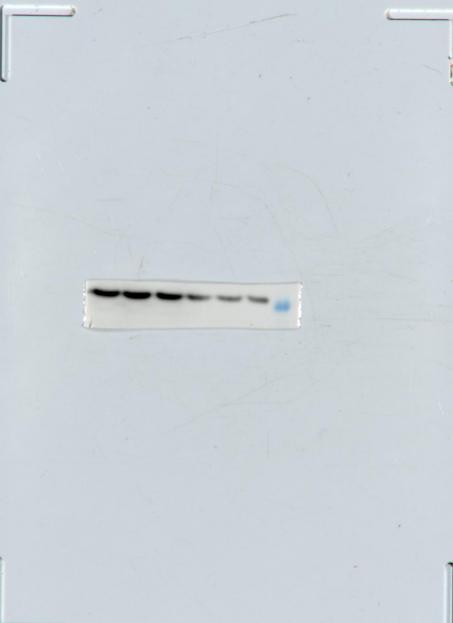


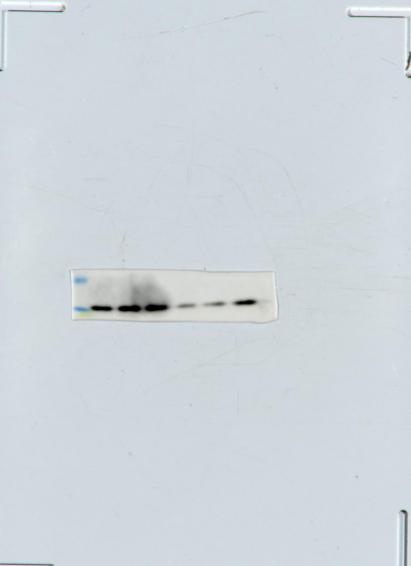

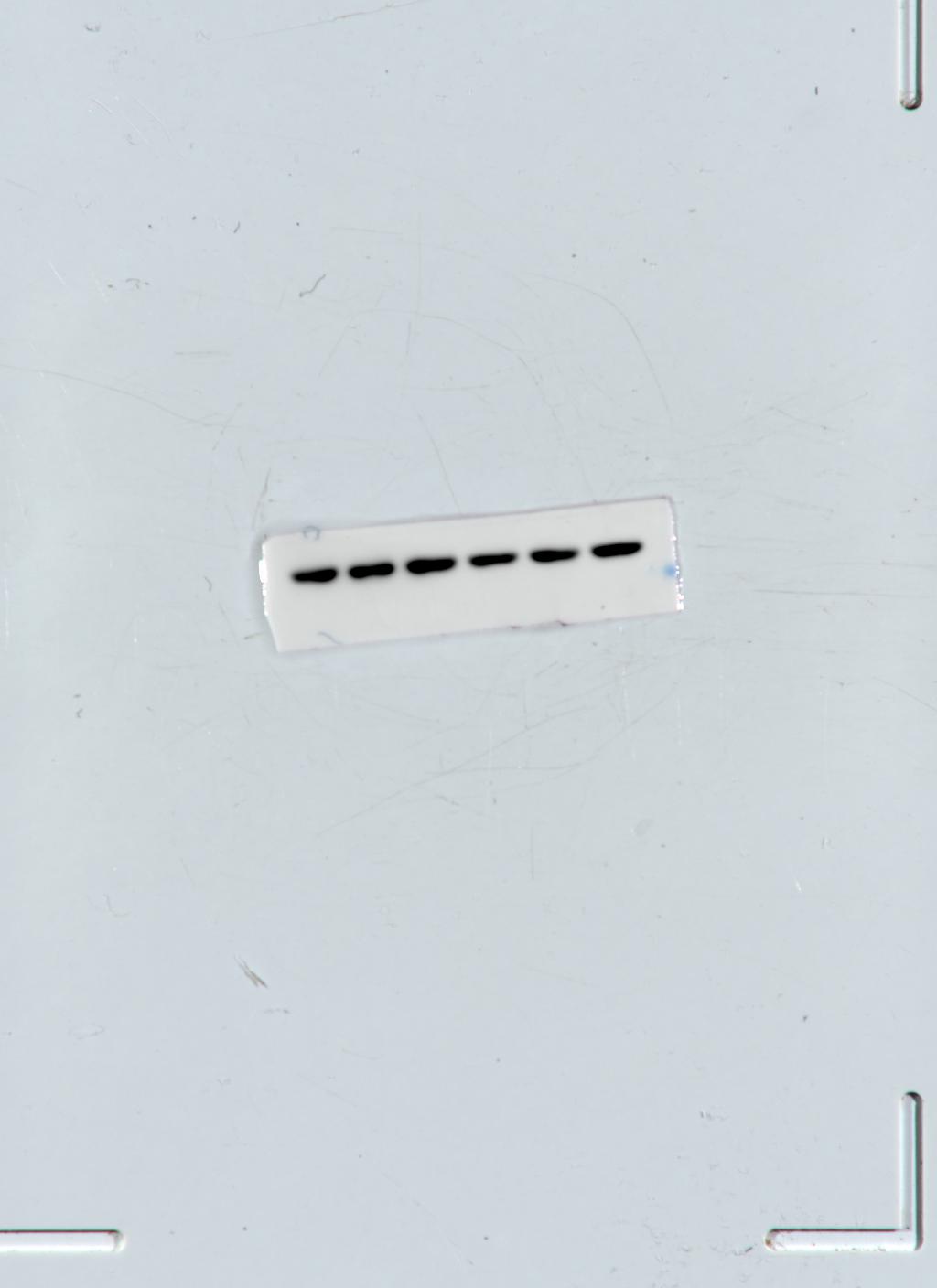


FIGURE 2I


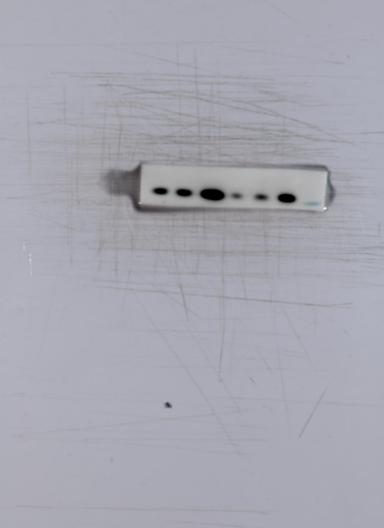


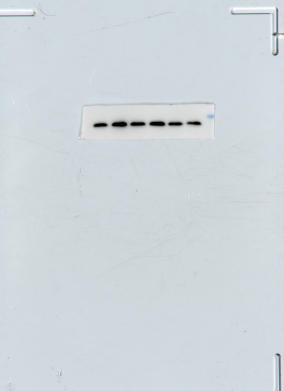

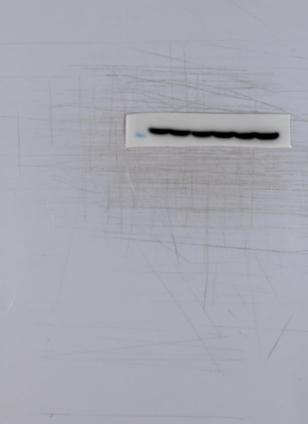


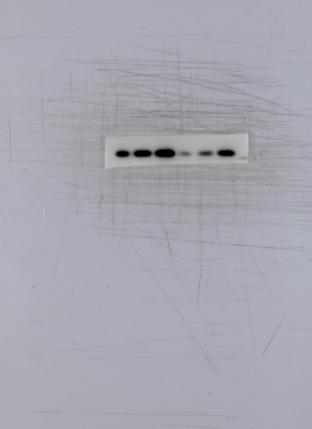


FIGURE 2J


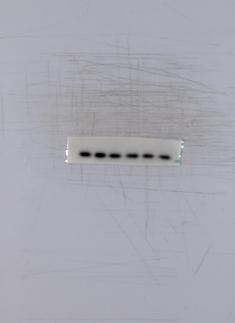

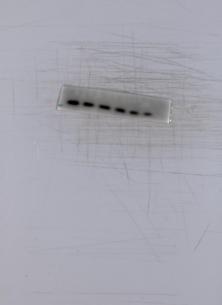


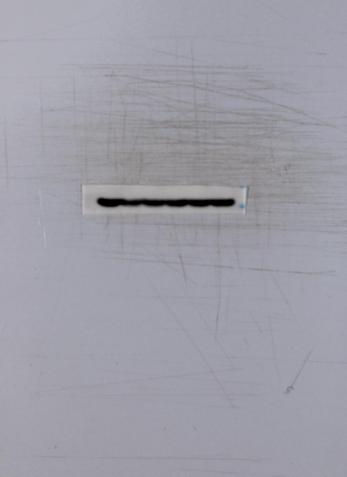

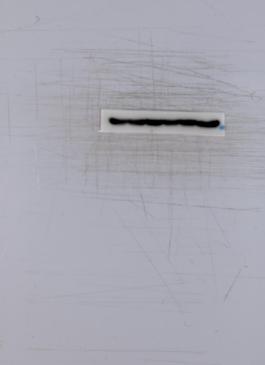


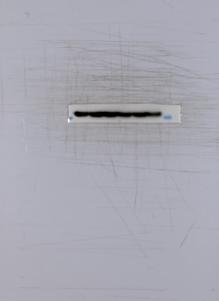


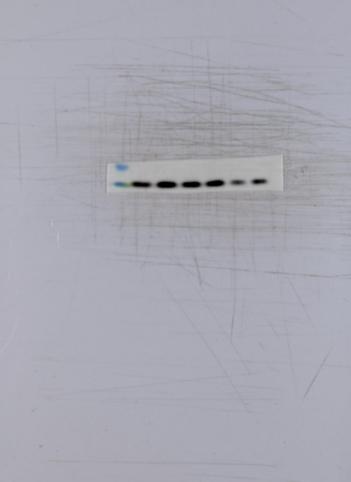

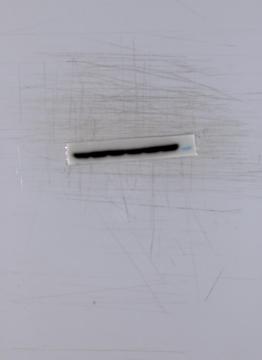


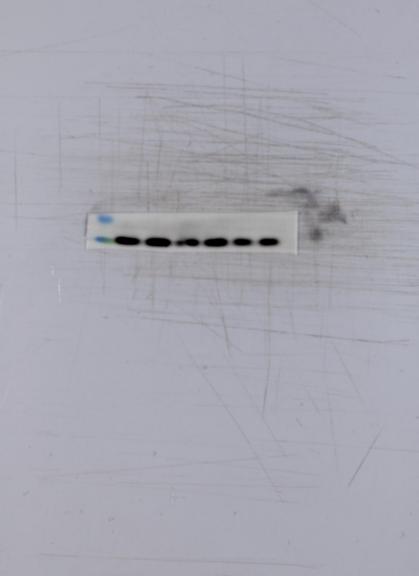


FIGURE 3C


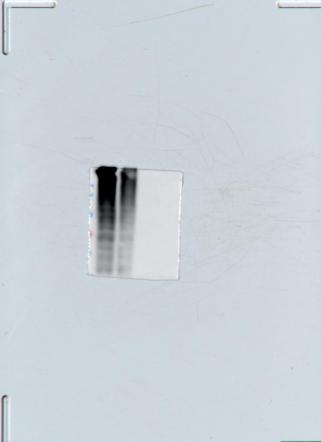

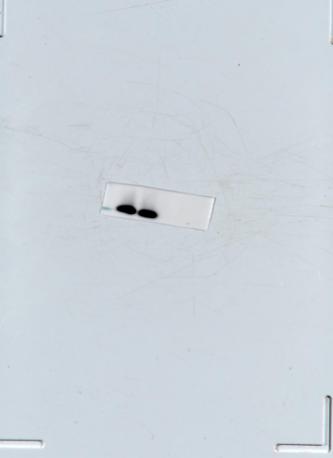


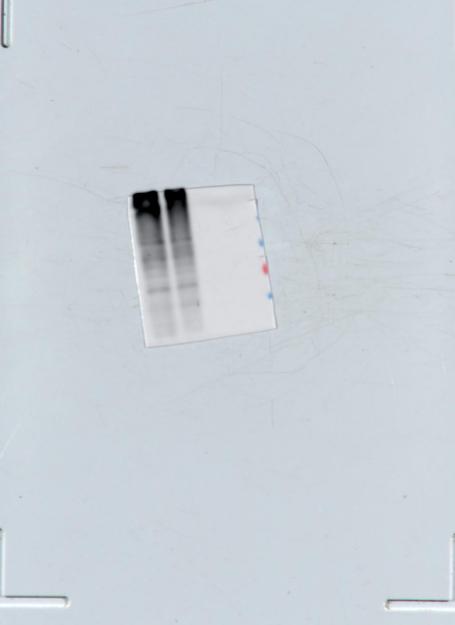


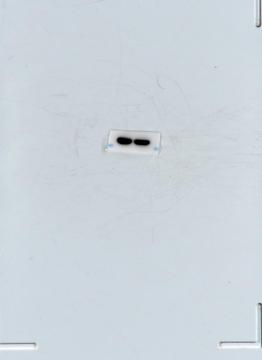


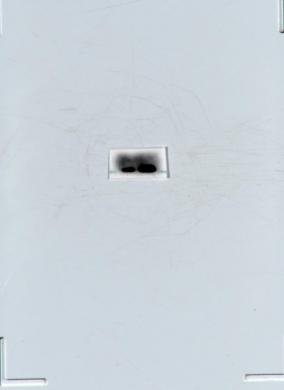


FIGURE 3D


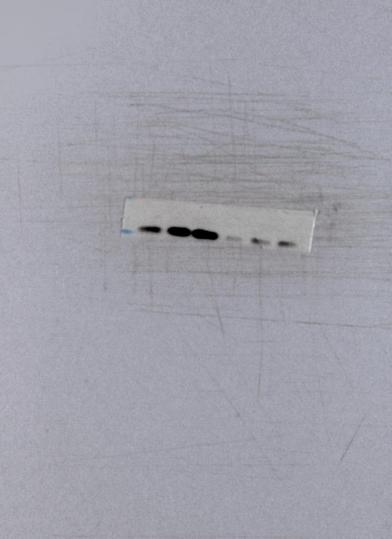

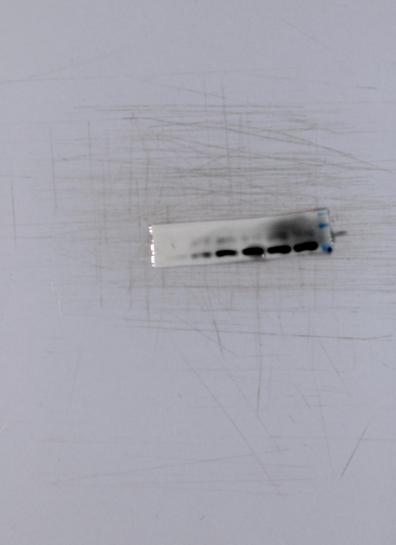

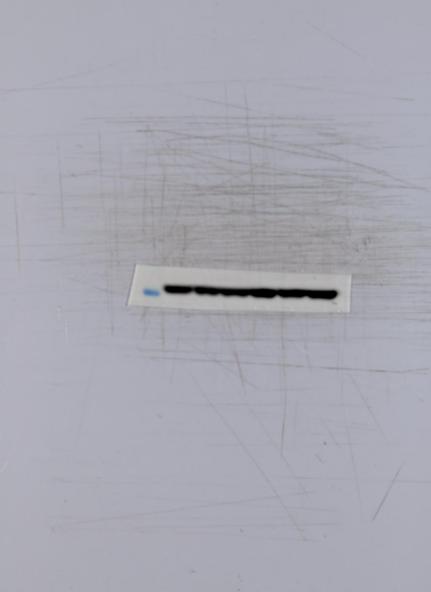


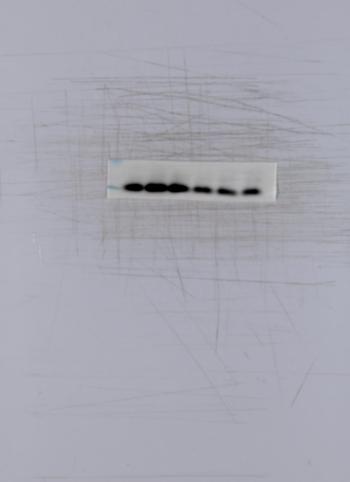

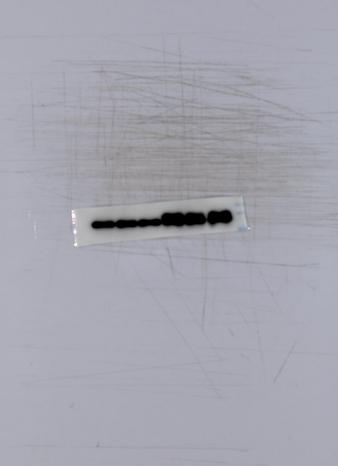


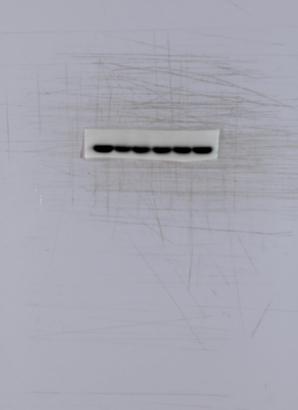


FIGURE 3E


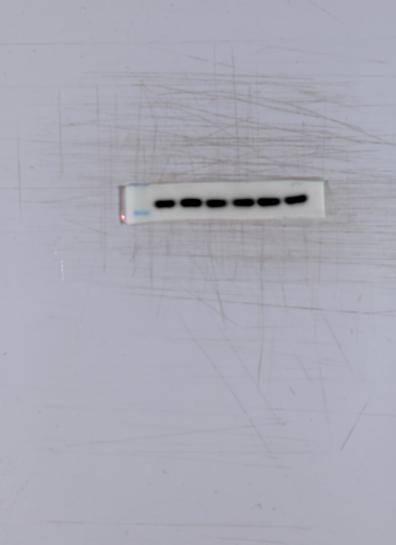


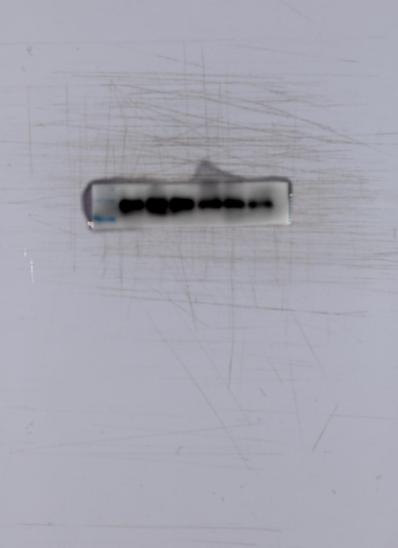

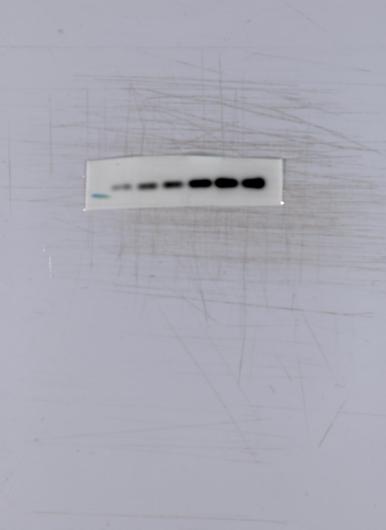


FIGURE 3F

FIGURE 3G

FIGURE 3H

FIGURE 4A

FIGURE 4B

FIGURE 4C

FIGURE 4D

FIGURE 4E

FIGURE 4F

FIGURE 4G

FIGURE 5A

FIGURE 5B

FIGURE 5C

FIGURE 5D

FIGURE 5E

FIGURE 5F

FIGURE 5G
